# Transcriptomic and chemogenomic analyses unveil the essential role of Com2-regulon in response and tolerance of *Saccharomyces cerevisiae* to stress induced by sulfur dioxide

**DOI:** 10.15698/mic2019.11.697

**Published:** 2019-09-30

**Authors:** Patrícia Lage, Belém Sampaio-Marques, Paula Ludovico, Nuno P. Mira, Ana Mendes-Ferreira

**Affiliations:** 1 Universidade de Trás-os-Montes e Alto Douro, Escola de Ciências da Vida e Ambiente; Vila Real, Portugal.; 2 BioISI - Biosystems & Integrative Sciences Institute, Campo Grande, Lisboa, Portugal.; 3 Life and Health Sciences Research Institute (ICVS), School of Medicine, University of Minho, 4710-057 Braga, Portugal.; 4 ICVS/3B's - PT Government Associate Laboratory, Braga/Guimarães, Portugal.; 5 Institute for Bioengineering and Biosciences, Instituto Superior Técnico, Department of Bioengineering, University of Lisbon, Portugal.

**Keywords:** Sulfur dioxide tolerance, Com2 (YER130c), wine preservation, Saccharomyces cerevisiae, stress response

## Abstract

During vinification *Saccharomyces cerevisiae* cells are frequently exposed to high concentrations of sulfur dioxide (SO_2_) that is used to avoid overgrowth of unwanted bacteria or fungi present in the must. Up to now the characterization of the molecular mechanisms by which *S. cerevisiae* responds and tolerates SO_2_ was focused on the role of the sulfite efflux pump Ssu1 and investigation on the involvement of other players has been scarce, especially at a genome-wide level. In this work, we uncovered the essential role of the poorly characterized transcription factor Com2 in tolerance and response of *S. cerevisiae* to stress induced by SO_2_ at the enologically relevant pH of 3.5. Transcriptomic analysis revealed that Com2 controls, directly or indirectly, the expression of more than 80% of the genes activated by SO_2_, a percentage much higher than the one that could be attributed to any other stress-responsive transcription factor. Large-scale phenotyping of the yeast haploid mutant collection led to the identification of 50 Com2-targets contributing to the protection against SO_2_ including all the genes that compose the sulfate reduction pathway (*MET3, MET14, MET16, MET5, MET10*) and the majority of the genes required for biosynthesis of lysine (*LYS2, LYS21, LYS20, LYS14, LYS4, LYS5, LYS1 and LYS9*) or arginine (*ARG5,6, ARG4, ARG2, ARG3, ARG7, ARG8, ORT1* and *CPA1*). Other uncovered determinants of resistance to SO_2_ (not under the control of Com2) included genes required for function and assembly of the vacuolar proton pump and enzymes of the antioxidant defense, consistent with the observed cytosolic and mitochondrial accumulation of reactive oxygen species in SO_2_-stressed yeast cells.

## INTRODUCTION

Sulfur dioxide (often abbreviated to sulfite or SO_2_) has been long used in winemaking due to its recognized potential in inhibiting growth of spoilage microbes whose activity decreases wine quality by producing off-flavor compounds (e.g. H_2_S and other sulfur-based volatiles) and causing formation of sediments or gas after bottling, among other deleterious effects. SO_2_ is usually added to the must in the form of potassium or sodium metabisulfite that, in solution, has a pH dependent speciation. At low pH the more abundant species is molecular SO_2_ (p*Ka* ~ 1.8), however, at the pH of wine (between 3 and 3.8) bisulfite (HSO_3_^−^; p*Ka* 6.9) is the more abundant form [1]. The antimicrobial potential of SO_2_ is believed to result from its ability to permeate the microbial plasma membrane by passive diffusion [2], similar to what is known to occur with carboxylic weak organic acids also used as preservatives (e.g. acetic or propionic acids) [3]. Once inside microbial cells molecular SO_2_ dissociates into bisulfite (HSO_3_^−^) and sulfite (SO_3_^2-^) due to the internal pH surpassing the pKa values [1]. The deleterious effects caused by accumulation of SO_2_, HSO_3_^−^ or SO_3_^2-^ inside *Saccharomyces cerevisiae* cells were described to include depletion of ATP caused by inhibition of glyceraldeyde-3-phosphate dehydrogenase and alcohol dehydrogenases [4], perturbation of the plasma membrane structure and damaging of proteins, vitamins or coenzymes due to cleavage of disulfide bonds [5, 6]. Although significant strain-to-strain variability is observed concerning tolerance to SO_2_, studies have identified spoilage yeast strains able to grow in the presence of concentrations as high as 600 mg/L [7–9], well above the levels legally permitted in enology which are in the range of 150 to 400 mg/L [10]. Among the more feared SO_2_-tolerant species are the yeasts *Saccharomycodes ludwigii* and *Brettanomyces bruxellensis*, with the first being particularly problematic due to the difficulties in eradicating it from contaminated environments using currently available sanitation methodologies [11]. As a response to that emergence in resilience to SO_2_ within spoilage species, wine producers tend to increase the concentration of this preservative. This practice has, however, adverse effects in health of more susceptible consumers [12, 13], while also rendering the wines less attractive in a market that demands “chemical-free” products and increasing pressure for selection of more tolerant strains.

Wine *S. cerevisiae* strains are considerably more tolerant to SO_2_ than laboratory strains and genomic analysis revealed that, in most cases, this phenotype results from a higher transcription of the *SSU1* gene [14–19], encoding a plasma membrane pump essential for efflux of sulfite and bisulfite [20]. This increased transcription of *SSU1* observed in SO_2_-tolerant wine strains results from chromosomal rearrangements that exchange the native promoter for a stronger one [14–18]. The more common of these rearrangements is a translocation between chromosome XVI (where the *SSU1* allele is natively located) and chromosome VIII, placing *SSU1* under the regulation of the strong promoter of *ECM34* [14, 15, 17]. Notably, *SSU1* transcription is not responsive to SO_2_ [16, 18] and thus the different levels of tolerance in *S. cerevisiae* wine strains are, in general, explained by the basal transcript levels of *SSU1* [16, 18]. An exception to this was observed for the 71B commercial strain, which was found to encode a SO_2_-responsive *SSU1* allele, presumably due to the retention of the original *SSU1* promoter in addition to two copies of *SSU1*-R [21]. The transcription factor Fzf1 is the regulator of *SSU1* expression from its endogenous promoter [20], however, it plays no role in the control of the higher expression of more tolerant strains since it has no binding site in the modified *SSU1* promoters these strains harbor [15, 18]. Besides the efflux of sulfite mediated by Ssu1, the production of acetaldehyde, the increased activity of the transulfuration pathway and the blockage of adenine biosynthesis have also been pinpointed as mechanisms contributing to the improved tolerance of *S. cerevisiae* to SO_2_ [22–24].

In this work, we demonstrate that the poorly characterized transcription factor Com2 (ORF YER130c) is essential for tolerance and response of *S. cerevisiae* cells to SO_2_ at pH 3.5. Com2 encodes an orphan homologue of the environmental stress-responsive transcription factors Msn2 and Msn4 [25], being derived from a separate locus in the ancestral yeast genome that existed before whole genome duplication [26]. Com2 was also found to be an orthologue of *Candida albicans* Mnl1, a species also harboring an orthologue of *S. cerevisiae* Msn4 but not of Msn2 [26]. CaMsn4 and CaMnl1 could not be implicated in a general environmental stress response in *C. albicans* [26] but CaMnl1 was found to mediate tolerance and response to high concentrations of acetic acid [26]. Although transcription of Com2 was found to increase in *S. cerevisiae* cells exposed to acetic, propionic or benzoic acids (at pH 4) [27–29], no protective effect against these acids could be attributed to this regulator [27–29]. By exploring a combination of transcriptomics and genome-wide phenotypic analyses, a detailed mechanism describing how Com2-dependent regulon contributes for tolerance and response of *S. cerevisiae* to SO_2_ is established in this study. The results also provided, for the first time, a genome-wide view of the *S. cerevisiae* genes required for maximal tolerance to SO_2_ at a low pH broadening the current view into the modes of toxicity of this preservative and underlying protective responses.

## RESULTS

### The transcription factor Com2 is a determinant of *S. cerevisiae* tolerance to SO_2_

**Figure 1:**
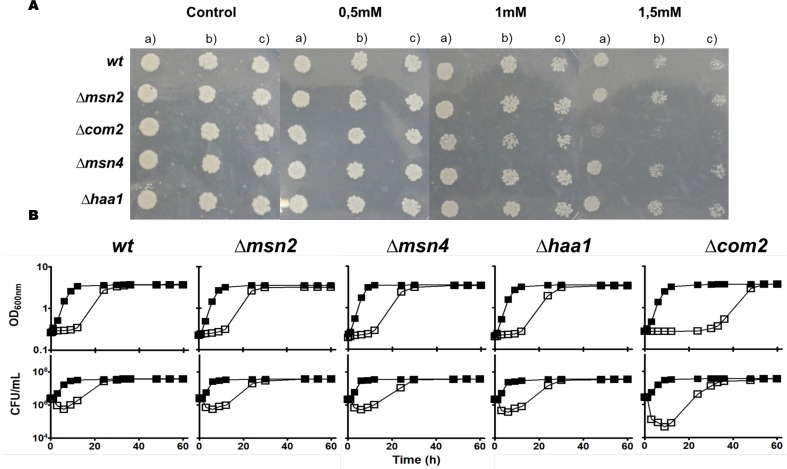
FIGURE 1. Comparison of the susceptibility to SO_2_ of the *Saccharomyces cerevisiae* BY4741 parental strain and the deletion mutants Δ*msn2*, Δ*msn4*, Δ*haa1* and Δc*om2* by spot assays **(A)** or by cultivation in MMB liquid medium (pH 3.5) (▪) or in this medium supplemented with 0.5 mM of SO2 (□) **(B)**. In (A) the cells used to prepare the spots were grown in MMB liquid medium until mid-exponential phase and then inoculated (at an OD of 0.05) in MMB (pH 3.5) agarized medium supplemented with the indicated concentrations of SO2. Lanes (b) and (c) are, respectively, 1:5 and 1:10 dilutions of the suspension used in lane (a). In (B) growth was followed by measuring culture OD_600_ and the concentration of viable cells was assessed as the number of colony forming units per ml of cell culture (CFU ml-1). All the results presented are representative of at least three independent experiments that gave the same pattern of results.

The demonstrated similarity of Com2 with Msn2/Msn4 [26] and the reported involvement of the latter two regulators in *S. cerevisiae* response to environmental stress, specifically to stress induced by carboxylic acids [30], prompted us to compare the susceptibility of mutants devoid of *COM2, MSN2* or *MSN4* to SO_2_ (at pH 3.5) with the one of the parental strain BY4741 **(Fig. 1)**. Since Haa1 was also implicated in tolerance to hydrophilic organic acids [27] and is a positive regulator of Com2 [28, 29], a mutant devoid of this gene was also included in this phenotypic profiling **(Fig. 1)**. Among the strains examined only *com2*Δ showed strong susceptibility to SO_2_ (at pH 3.5), this being visible both in solid and in liquid medium **(Fig. 1A)**. Consistently, cellular viability of SO_2_-challenged *com2*Δ cells was well below the one registered for any of the other strains, this difference being particularly noticeable during the adaptation period **(Fig. 1B)**. These results suggest that Com2 plays an essential role in the early adaptation phase of *S. cerevisiae* to stress imposed by SO_2_ at pH of 3.5. We tested whether deletion of Com2 would provide tolerance to other stresses including toxic concentrations of ethanol, copper or of H_2_O_2_ with no significant protective effect being observed (results not shown).

### General overview on the yeast transcriptional response to SO_2_ in BY4741 and in BY4741_*com2*Δ cells

The identification of Com2 as a new determinant of yeast tolerance to SO_2_ led us to investigate its role in the transcriptome-wide alterations imposed by stressful concentrations of that preservative. For this, we have resorted to a transcriptomic profiling of *S. cerevisiae* BY4741 and *com2*Δ cells cultivated for 60 min in the absence or presence of 0.5 mM SO_2_ (at pH 3.5), these being experimental conditions comparable to those used in the susceptibility assays shown in **Fig. 1**. The selected time-point for the transcriptomic analysis corresponds to the early phase of adaptation to SO_2_ (Figure S1), a period where strong adaptive responses to carboxylic acid-induced stresses have been observed [28, 29] and where Com2 seems to exert a more prominent protective effect **(Fig. 1B)**.

Principal component analysis of the results obtained for the different samples used in the transcriptomic analysis revealed that exposure to SO_2_ greatly impacted genomic expression in both wild-type and *com2*Δ yeast cells, although this effect was markedly different for the two strains (Figure S1). Comparison of the transcriptomes of wild-type and *com2*Δ cells grown in the absence of SO_2_ (control conditions) revealed only 48 genes with significantly different expression in the two strains, 28 of these being more expressed (above 2-fold) in the wild-type strain and 20 being more expressed in the mutant strain (using the same 2-fold threshold). The list of these genes affected upon *COM2* deletion in control conditions is provided in Table S1.

**Figure 2:**
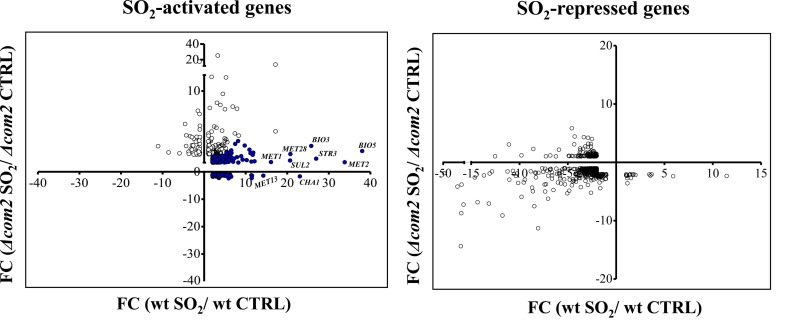
FIGURE 2: Overview on the transcriptomic profile of SO_2_-challenged BY4741 or Δcom2 cells. The genes whose transcription increased more than 2-fold in the presence of SO_2_, comparing with the levels attained in control conditions, were selected for this representation. Genes activated by SO_2_ in wild-type cells but not in the mutant were considered as Com2-targets and are herein highlighted in blue.

Exposure to SO_2_ resulted in a dramatic change in the genomic expression of BY4741 cells with 569 genes being up-regulated and 456 genes down-regulated, comparing with the levels attained in control conditions (listed in Table S2). In the *com2*Δ strain, exposure to SO_2_ resulted in the activation of 161 genes and in repression of 242, however, these genes had little overlap with those that changed their expression in the wild-type strain (see data and figure in Table S3). The differences in the genomic expression programs prompted by both strains in response to SO_2_ are rendered clear in **Fig. 2**. A high number of genes activated by SO_2_ in wild-type cells were not over-expressed in the mutant background and, in some cases, these genes were even repressed **(Fig. 2)**. These SO_2_-responsive genes lacking activation in the mutant are highlighted in blue in **Fig. 2** and were considered as Com2-targets. In concerns to genes down-regulated by SO_2_, a higher similarity was observed for wild-type and *com2*Δ cells, although it was still possible to observe genes that were down-regulated in the wild-type but not in the mutant and vice versa **(Fig. 2)**. However, because Com2 is predicted as a transcriptional activator, we did not consider the genes differently repressed by SO_2_ in the wild-type and in the *com2*Δ mutant as being Com2-targets.

To render the toxic effects of SO_2_ at a low pH in the physiology of *S. cerevisiae* BY4741 cells clearer, the genes found to be up- and down-regulated were clustered according with their predicted biological function using the MIPS functional catalogue. The biological functions enriched in the dataset of SO_2_-induced genes are “amino acid metabolism”, “nitrogen and sulfur metabolism”, “metabolism of vitamins and prosthetic groups”, “ion transport”, “amino acid transport” and “interaction with the environment”, as detailed in Figure S2. In general, these functional classes coincide with those obtained in the prior transcriptomics analyses of *S. cerevisiae* SO_2_-stressed cells [31, 32] and also of *C. albicans* [33]. Despite this, our number of SO_2_-responsive genes was much higher than the one reported in other studies performed in *S. cerevisiae* [31, 32], which can be attributed to the higher sensitivity of the BY4741 strain in comparison with the susceptibility of the wine strains explored in these other studies [31, 32].

**Figure 3:**
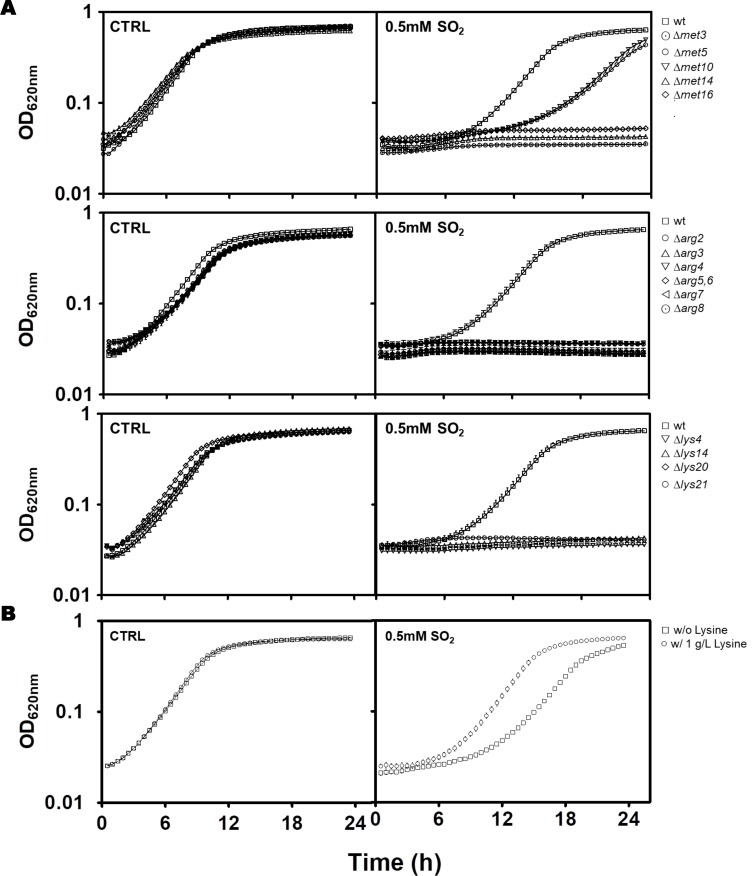
FIGURE 3. **(A)** Comparison of the susceptibility towards SO_2_ of the mutants devoid of the expression of genes whose transcription is activated in response to SO_2_ in a Com2p-dependent manner. Cells of the parental strain (wt) and of the indicated deletion mutants were grown until mid-exponential phase in liquid MMB medium (at pH 3.5) and then used to inoculate the same basal medium either supplemented (open symbols) or not supplemented with 0.5 mM SO_2_ (filled symbols) (at pH 3.5). Cells were batch cultured at 30ºC and growth was monitored based on OD_620nm_. The growth curves presented are representative of at least three independent growth experiments. **(B)** Wild-type strain was challenged for 24h in MMB pH 3.5 without and/or with 0.5 mM SO_2_ supplemented with 1 g/L of lysine.

A closer look into the data revealed that the “amino acid metabolism” and “nitrogen and sulfur metabolism” classes were largely composed of genes involved in the sulfate assimilation pathway (*SUL1, SUL2, MET3, MET14, MET16, MET5, MET10*) as well as genes involved in biosynthesis of lysine (*LYS20, LYS21, LYS4, LYS2, LYS9* and *LYS1*) and arginine (*ARG2, ARG3, ARG7, ARG4, ARG5/6, CPA1* and *ARG81*) (Table S1). Consistent with their observed up-regulation, deletion of *MET3, MET5, MET10, MET14, MET16, LYS4, LYS14, LYS20, LYS21, ARG2, ARG3, ARG4, ARG5, ARG7* genes led to a strong susceptibility phenotype to SO_2_ at pH 3.5 **(Fig. 3)**. Previously, the addition of methionine to the medium was found to increase susceptibility of yeast cells to SO_2_ by inhibiting the activity of the sulfate assimilation pathway [22]. Under the conditions that we have tested this does not seem to be the case since susceptibility to SO_2_ of the BY4741 strain (auxotrophic for methionine) was identical to the one of the prototrophic yfg*Δ*0 strain which was cultivated in a growth medium not supplemented with methionine (compare results in Figures.S1, S3 and Fig. 1). Furthermore, the susceptibility phenotype of the *met5*Δ, *met14*Δ and *met16*Δ mutants to SO_2_ was still clearly detectable in the prototrophic background (Figure S3). Supplementation of the MMB medium with lysine (1.0 g/L) alleviated SO_2_ toxicity **(Fig. 3)**, while in the case of arginine a positive effect was also observed but only for the highly susceptible strain *com2*Δ (results not shown).

Consistent with the reported lack of transcriptional regulation of *SSU1* by SO_2_ in *S. cerevisiae* [15, 31, 32], we could not detect the up-regulation of this sulfite exporter neither in the transcriptomic profiling performed nor in other additional experiments that were performed using different cell extracts (Figure S4). Genes involved in acetaldehyde synthesis were also not found to be activated in response to SO_2_, despite the description that increased synthesis of this sulfur-sequestering carbohydrate ameliorate the toxic effects of SO_2_ [23, 24]. Nonetheless, reduced expression of genes encoding enzymes involved in degradation of acetaldehyde into ethanol or acetate was detected **(Table 1)**, which could indicate an attempt to increase (or at least maintain) the internal concentration of acetaldehyde [24].

**TABLE 1.** List of genes whose SO2-induced transcriptional activation registered in S. cerevisiae BY4741 cells is abolished in the absence of COM2. Genes whose elimination leads to an increase in yeast susceptibility to SO2 are highlighted in gray.

**Gene name**	$FC\left(\frac{wt\;\;SO2}{wt\;\;CTRL}\right)$	$FC\left(\frac{\Delta com2\;\;SO2}{\Delta com2\;\;CTRL}\right)$	**Function**
ARG3	5.18	1.05	Ornithine carbamoyltransferase (carbamoylphosphate:L-ornithine carbamoyltransferase), catalyzes the sixth step in the biosynthesis of the arginine precursor ornithine.
ARG7	2.67	1.19	Mitochondrial ornithine acetyltransferase, catalyzes the fifth step in arginine biosynthesis; also possesses acetylglutamate synthase activity, regenerates acetylglutamate while forming ornithine.
ARG8	4.31	−1.05	Acetylornithine aminotransferase, catalyzes the fourth step in the biosynthesis of the arginine precursor ornithine.
ARO8	2.19	−1.02	Aromatic aminotransferase I, expression is regulated by general control of amino acid biosynthesis
ARO9	3.09	1.12	Aromatic aminotransferase II, catalyzes the first step of tryptophan, phenylalanine, and tyrosine catabolism
BAS1	2.83	1.25	Myb-related transcription factor involved in regulating basal and induced expression of genes of the purine and histidine biosynthesis pathways; also involved in regulation of meiotic recombination at specific genes
BIO2	11.82	2.26	Biotin synthase, catalyzes the conversion of dethiobiotin to biotin, which is the last step of the biotin biosynthesis pathway; complements E. coli bioB mutant.
BIO3	25.77	3.1	7,8-diamino-pelargonic acid aminotransferase (DAPA), catalyzes the second step in the biotin biosynthesis pathway; BIO3 is in a cluster of 3 genes (BIO3, BIO4, and BIO5) that mediate biotin synthesis.
BIO4	10.23	1.32	Dethiobiotin synthetase, catalyzes the third step in the biotin biosynthesis pathway; BIO4 is in a cluster of 3 genes (BIO3, BIO4, and BIO5) that mediate biotin synthesis; expression appears to be repressed at low iron levels.
DAL3	9.76	3.15	Ureidoglycolate hydrolase, converts ureidoglycolate to glyoxylate and urea in the third step of allantoin degradation; expression sensitive to nitrogen catabolite repression
DAL82	3.01	1.56	Positive regulator of allophanate inducible genes; binds a dodecanucleotide sequence upstream of all genes that are induced by allophanate; contains an UISALL DNA-binding, a transcriptional activation, and a coiled-coil domain
DBF2	4.24	1.31	Ser/Thr kinase involved in transcription and stress response; functions as part of a network of genes in exit from mitosis; localization is cell cycle regulated; activated by Cdc15p during the exit from mitosis; also plays a role in regulating the stability of SWI5 and CLB2 mRNAs
EEB1	4.87	1.31	Acyl-coenzymeA:ethanol O-acyltransferase responsible for the major part of medium-chain fatty acid ethyl ester biosynthesis during fermentation; possesses short-chain esterase activity; may be involved in lipid metabolism and detoxification
EHT1	4.57	−1.09	Acyl-coenzymeA:ethanol O-acyltransferase that plays a minor role in medium-chain fatty acid ethyl ester biosynthesis; possesses short-chain esterase activity; localizes to lipid particles and the mitochondrial outer membrane
ILV3	2.36	−1.06	Dihydroxyacid dehydratase, catalyzes third step in the common pathway leading to biosynthesis of branched-chain amino acids
ILV5	2.07	1.04	Bifunctional acetohydroxyacid reductoisomerase and mtDNA binding protein; involved in branched-chain amino acid biosynthesis and maintenance of wild-type mitochondrial DNA; found in mitochondrial nucleoids
LEU1	3.07	1.09	Isopropylmalate isomerase, catalyzes the second step in the leucine biosynthesis pathway
LEU4	2.5	1.19	Alpha-isopropylmalate synthase (2-isopropylmalate synthase); the main isozyme responsible for the first step in the leucine biosynthesis pathway
LEU9	2.14	1.14	Alpha-isopropylmalate synthase II (2-isopropylmalate synthase), catalyzes the first step in the leucine biosynthesis pathway; the minor isozyme, responsible for the residual alpha-IPMS activity detected in a leu4 null mutant
LYS1	4.14	1.16	Saccharopine dehydrogenase (NAD+, L-lysine-forming), catalyzes the conversion of saccharopine to L-lysine, which is the final step in the lysine biosynthesis pathway; also has mRNA binding activity.
LYS4	3.03	−1.25	Homoaconitase, catalyzes the conversion of homocitrate to homoisocitrate, which is a step in the lysine biosynthesis pathway.
LYS9	3.26	−1.11	Saccharopine dehydrogenase (NADP+, L-glutamate-forming); catalyzes the formation of saccharopine from alpha-aminoadipate 6-semialdehyde, the seventh step in lysine biosynthesis pathway; exhibits genetic and physical interactions with TRM112.
MET14	3.44	−2.07	Adenylylsulfate kinase, required for sulfate assimilation and involved in methionine metabolism
MET16	11.37	1.13	3′-phosphoadenylsulfate reductase, reduces 3′-phosphoadenylyl sulfate to adenosine-3′,5′-bisphosphate and free sulfite using reduced thioredoxin as cosubstrate, involved in sulfate assimilation and methionine metabolism
MET22	3.45	1.27	Bisphosphate-3′-nucleotidase, involved in salt tolerance and methionine biogenesis; dephosphorylates 3′-phosphoadenosine-5′-phosphate and 3′-phosphoadenosine-5′-phosphosulfate, intermediates of the sulfate assimilation pathway
MET3	12.13	1.17	ATP sulfurylase, catalyzes the primary step of intracellular sulfate activation, essential for assimilatory reduction of sulfate to sulfide, involved in methionine metabolism.
MET8	4.88	−1.41	Bifunctional dehydrogenase and ferrochelatase, involved in the biosynthesis of siroheme, a prosthetic group used by sulfite reductase; required for sulfate assimilation and methionine biosynthesis.
STP22	2.25	1.02	Component of the ESCRT-I complex, which is involved in ubiquitin-dependent sorting of proteins into the endosome; homologous to the mouse and human Tsg101 tumor susceptibility gene; mutants exhibit a Class E Vps phenotype
SUL1	11.44	−2.33	High affinity sulfate permease of the SulP anion transporter family; sulfate uptake is mediated by specific sulfate transporters Sul1p and Sul2p, which control the concentration of endogenous activated sulfate intermediates.
SUL2	20.67	1.27	High affinity sulfate permease; sulfate uptake is mediated by specific sulfate transporters Sul1p and Sul2p, which control the concentration of endogenous activated sulfate intermediates.
URE2	2.2	1.15	Nitrogen catabolite repression transcriptional regulator that acts by inhibition of GLN3 transcription in good nitrogen source; has glutathione peroxidase activity and can mutate to acquire GST activity; altered form creates [URE3] prion
VAC17	3.82	−1.38	Phosphoprotein involved in vacuole inheritance; degraded in late M phase of the cell cycle; acts as a vacuole-specific receptor for myosin Myo2p
VPS9	2.47	1.35	A guanine nucleotide exchange factor involved in vesicle-mediated vacuolar protein transport; specifically stimulates the intrinsic guanine nucleotide exchange activity of Vps21p/Rab5: similar to mammalian ras inhibitors; binds ubiquitin
ZAP1	4.33	1.56	Zinc-regulated transcription factor; binds to zinc-responsive promoters to induce transcription of certain genes in presence of zinc, represses other genes in low zinc; regulates its own transcription; contains seven zinc-finger domains

### Defining the Com2-regulon in response to SO_2_

Deletion of *COM2* abolished the transcriptional activation of 503 genes (these genes being highlighted in blue in **Fig. 2)** and reduced, by more than 50%, the SO_2_-induced activation of other 15 genes. It is important to highlight that although a short-exposure time to SO_2_ had been used to identify the Com2-regulated genes, more specifically responding to that preservative, it is likely that this high number of genes might not result because they are specific targets of Com2 in response to SO2 but rather because they reflect a different physiological state of the much more susceptible mutant cells, in comparison with the wild-type cells. A subset of these SO_2_-induced genes up-regulated by Com2 is shown in **Table 1** and the full list is provided in Table S4. From the functional point of view, this set of Com2-targets was highly enriched for genes involved in sulfate assimilation (*MET8, MET10, MET3, MET5, MET14, MET1, MET22* and *MET16*) and transport (*SUL1* and *SUL2*), as well as other genes associated with methionine and cysteine metabolism and regulation (*MET32, SAM2, HOM3, MET13 MET1, MHT1, SAM1, MET4, STR2, MET30* and *STR3*). Also included is this regulon are genes involved in biosynthesis of lysine (*LYS2, LYS21, LYS20, LYS14, LYS4, LYS5, LYS1 and LYS9*), arginine (*ARG5,6, ARG4, ARG2, ARG3, ARG7, ARG8, ORT1* and *CPA1*) or biotin (*BIO2, VHR1, ISA1, BIO5, BIO4* and *BIO3*), a sulfur containing vitamin. The positive effect of Com2 in up-regulating some of these SO_2_-responsive genes was confirmed by RT-PCR (Figure S4). Twelve of these genes up-regulated by Com2 under SO_2_ stress (*PHO5, SAM1, Ho, SAM2, USB1, OPT1, MET13, YHP1, MET6, PSD1, MOG1* and *WSC4*) already showed a lower expression in the mutant under control conditions, although the impact of *COM2* deletion in the expression of these genes is significantly higher in the presence of SO_2_ (Table S1).

### Genome-wide profiling of SO_2_-resistance genes: role of genes of the Com2-regulon

The strong susceptibility phenotype exhibited by *com2*Δ cells to SO_2_ may result from the reduced expression of genes required for maximal tolerance to that compound. Indeed, the protective effect against SO_2_ of the Com2-regulated genes *MET3, MET5, MET10, MET14, MET16, ARG2, ARG3, ARG4, ARG5/6, ARG7, ARG8, LYS4, LYS14, LYS20* and *LYS21* was demonstrated in this work **(Fig. 3A** and **B)**. However, the very large size of the Com2-regulon renders difficult to individualize the individual contribution of the remaining target genes in tolerance to SO_2_ and therefore we have resorted to the utilization of the yeast deletion mutant collection for a broader phenomic analysis. In the phenotypic profiling undertaken all the 5,000 haploid yeast strains that are devoid of all non-essential genes (generally known as the yeast disruptome) were profiled for their growth in MMB solid medium supplemented with 1, 1.5 or 2 mM of SO_2_ (at pH 3.5). This screening revealed around 767 mutants with reduced growth in the presence of SO_2_, comparing with the one exhibited by wild-type cells, 282 being hyper-susceptible (unable to grow at 1 mM) and 485 susceptible (did not show growth at 1.5 mM or 2 mM) (Table S5). Around 100 of the identified SO_2_-susceptible strains are devoid of genes providing protection against multi-stresses in yeast (highlighted in grey in Table S5) [34] and therefore their identification in this screening was expected. Comparison of the identified SO_2_-resistance genes with those that were found to be transcriptionally activated in response to this chemical revealed a small overlap of only 50 genes (highlighted in grey in Table S5). This observation is in line with previous reports describing a poor correlation between yeast genes induced in response to environmental stressors (including to stress induced by carboxylic acids) and those providing protection against these stresses [3, 28, 29, 35]. Crossing the set of defined Com2 targets with the list of genes providing tolerance to SO_2_ resulted in the identification of 47 genes (highlighted in grey in Table S5 and in **Table 1)**. Besides the Com2-targets involved in biosynthesis of lysine and in the sulfate assimilation pathway that were mentioned above, the other Com2-targets contributing to SO_2_ tolerance uncovered by the phenomic analysis included genes involved in transcriptional regulation (e.g. *ZAP1, BAS1, URE2*), in regulation of cell cycle (e.g. *BUD14, BUD16, DMA2* or *KIP2*) or in transport (e.g. *MCH5, STP22* or *MDM34*).

**Figure 4:**
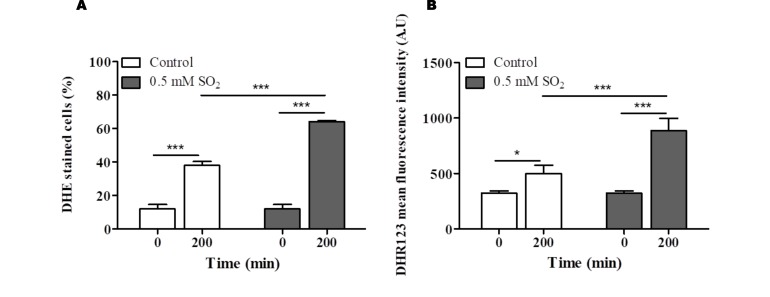
FIGURE 4. Assessment of the intracellular accumulation of ROS in *S. cerevisiae* BY4741 cells cultivated in MMB medium (at pH 3.5) (white bars) or in this same medium supplemented with 0.5 mM SO_2_ (grey bars). Quantification of intracellular ROS accumulation was made based on fluorescence emitted by cells stained with the ROS specific dyes DHE **(A)** and DHR123 **(B)** and analysed by flow cytometry. Significance was determined by two-way ANOVA (*p≤0.05, ***p≤0.001) between cells at time 0 and 200 minutes and between control cells and cells supplemented with SO_2_. Data represents mean ± SEM (the standard error of the mean) of at least three biological independent replicas.

Functional clustering of the determinants of SO_2_ resistance uncovered in this work (shown in Fig. S5) reinforced the relevance of genes involved in biosynthesis of arginine, methionine and lysine (see **Fig. 3)**, as well of genes required for biosynthesis of adenine (e.g. *BAS1, ADE2*), glycine, serine or histidine (e.g. *GVC1, SER33, SER1, SER2*) for maximal tolerance to this preservative (Table S5). Genes involved in synthesis of the reserve carbohydrates trehalose and glycogen (e.g. *GPH1, TSL1, TPS1, TPS2, TPS3*) as well as the key glycolytic enzymes Pfk1, Tdh3 and Tpi1 were also identified as being critical determinants of SO_2_ tolerance (Table S5 and Fig. S5), presumably by contributing to generate enough energy to allow the cells to activate the necessary defense mechanisms. Consistent with this idea, inactivation of glycolysis has been pinpointed as one of the relevant mechanisms of toxicity of sulfite in yeast [4] and genes required for assembly of components of the electron transfer (*COQ5, COX11, COX14, COX15* or *COX23*) and of F_1_F_0_-ATPase function (e.g *ATP1, ATP11, ATP12* or *ATP15*) were also found to provide protection against SO_2_ (supplementary Table S5). Another detectable feature was the identification of all genes required for assembly and function of the V-ATPase (*VMA1, VMA11, VMA16, VMA21, VMA3, VMA4, VMA5, VMA7* or *VPS1*) as being essential for tolerance to SO_2_ (Table S5 and Figure. S5). SO_2_ was found to induce intracellular acidification [3] and therefore the requirement for V-ATPase is likely to be related with the demonstrated involvement of this pump in restoring internal pH to physiological values upon acid-induced stress [2, 24]. Several genes involved in antioxidant defense were also found to provide protection against SO_2_ including the cytosolic superoxide dismutase *SOD1*, the peroxiredoxin *AHP1*, the reductases *GRX3* or *MXR2* and the glutathione biosynthetic enzyme *GSH1* (Table S5). These observations suggest that one of the features of SO_2_ exposure is the induction of oxidative stress, a hypothesis that was confirmed in SO_2_-challenged cells by the higher accumulation of reactive oxygen species (ROS) in the cytosol, as revealed by the higher fluorescence intensity levels of DHR123, and of the ROS superoxide anion, revealed by the higher number of cells stained with DHE **(Fig. 4)**. In line with these observations, a mutant devoid of cytosolic superoxide dismutase has been found to have increased susceptibility to sulfite (also in line with the results from our disruptome screening), this being hypothesized to result from an oxyradical-based mechanism of toxicity of this chemical [36]. Sulfite has been found to induce oxidative stress in kidney, cardiac and plant cells [37, 38], the mechanism behind this being hypothesized to result from the perturbation of mitochondrial function mainly through the inhibition of cytochrome c activity by sulfide accumulated upon sulfite reduction [37]. Several genes encoding components of the cytochrome c oxidase complex were found to have a reduced expression in response to SO_2_ in *S. cerevisiae*, with *CYC7* exhibiting a particularly prominent repression reaching almost 50-fold (Table S2).

## DISCUSSION

**Figure 5:**
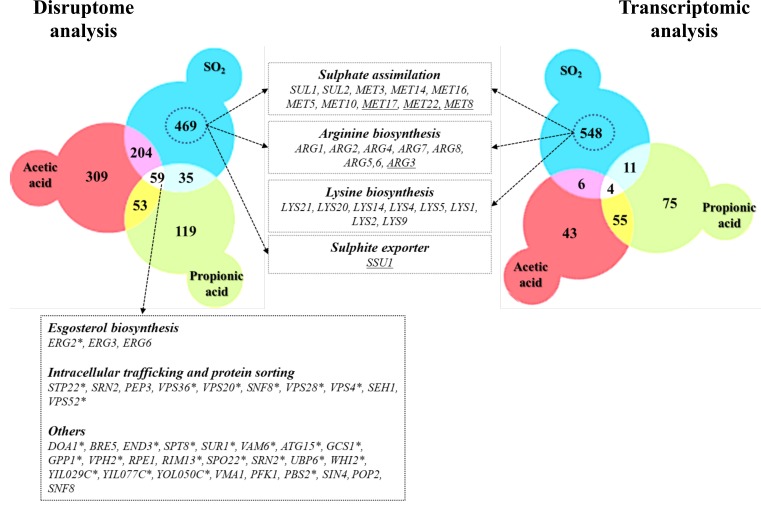
FIGURE 5. Venn diagrams comparing genes that were found to confer resistance to SO_2,_ propionic and acetic acids (left panel) and the set of genes up-regulated by SO_2_ with those responding to acetic or propionic acids (right panel). Genes with asterisk corresponds to the MDR (multidrug resistance) genes and underlined genes represents the identified SO_2_-resistance genes with those that were found to be transcriptionally activated in response to this chemical.

**Figure 6:**
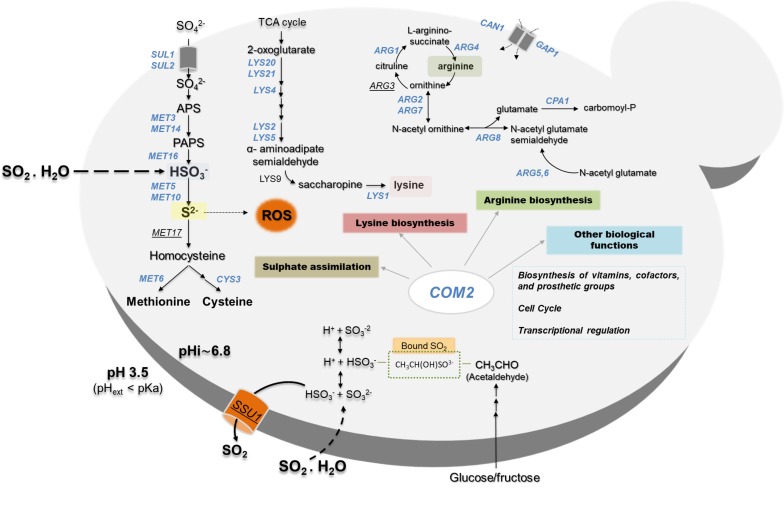
FIGURE 6. Schematic model for the adaptive response of *S. cerevisiae* to SO_2_ -induced stress according with the results obtained in the herein described transcriptomic and chemogenomic analyses and also integrating previously described adaptive responses. Genes regulated by Com2 are highlighted in blue.

In this work, the responses of *S. cerevisiae* cells to SO_2_ at the enological relevant pH of 3.5 were investigated integrating results from transcriptomic and chemogenomic analyses. Although the mode of action of SO_2_ was associated with the one of weak carboxylic organic acids [2, 24], comparison of the genes up-regulated by SO_2_ with those responding to acetic or propionic acids (chosen for this comparative analysis for being more hydrophilic, like sulfite) reveals a modest overlap **(Fig. 5)**. The set of genes found to provide protection against acetic or propionic acids also showed little similarity with those required for maximal tolerance to SO_2_ **(Fig. 5)** and SO_2_ induced the Com2-dependent regulon while acetic and propionic acids rely on the Haa1-dependent regulons [27–29]. Altogether, these observations suggest that once inside yeast cells, SO_2_, acetic and propionic acids exert dissimilar toxic effects and, consequently, the responses evolved by yeast cells are different and require different players. The fact that SO_2_ is not organic is likely to be on the basis of these anticipated toxic effects in the cellular environment. The genes of the sulfate activating pathway emerged among the SO_2_-specific transcriptional responses as well as among the SO_2_-specific determinants of resistance (SO_2_) **(Fig. 5)**. Prior studies have referred to the relevance of the sulfate activation pathway in yeast response to SO_2_ [22, 32], however, this was mostly based on the observed higher expression of these genes in more tolerant strains [22]. Interestingly, up-regulation of genes of the sulfate activation pathway in response to sulfite stress was observed in *C. albicans* and in *Vitis vinifera* [37, 38] indicating that responsiveness of this pathway to sulfite/SO_2_ stress is not unique to *S. cerevisiae*. In the present study, we not only confirmed that the transcription of genes of the sulfate activating pathway is highly responsive to SO_2_ stress, but we also showed that these genes are critical for tolerance. The protective effect of the sulfite reductases Met5 and Met10 goes in line with the anticipated involvement of these enzymes in reducing the intracellularly accumulated sulfite into sulfide [22, 32], which can then be channeled to synthesis of methionine and cysteine (as schematically represented in **Fig. 6)**. However, the requirement for Met3, Met14 or Met16 tolerance to SO_2_ is much less obvious since these enzymes are located upstream of the sulfite-sulfide reduction step **(Fig. 6)**. In plants the involvement of the sulfate reduction steps in response to sulfite was linked with an increase in intracellular sulfate caused by sulfite oxidation via a sulfite oxidase [37]. We cannot exclude oxidation of intracellularly accumulated sulfite into sulfate or another species (e.g. sulfonic acid), which could require detoxification through the sulfate activating pathway, however, we do not favor this hypothesis since sulfite oxidases were never described in *S. cerevisiae* (although it has been shown in *Rhodotorula* [39]). Sulfite was found to degrade thiamine [40] and thus one may hypothesize that it may also have a deleterious effect against other sulfur-containing molecules like cysteine, methionine or homocysteine. Under these conditions, SO_2_-stressed cells would require a high flux through the sulfate activating pathway as a compensatory response, something that could not be achieved in the *met3*Δ, *met14*Δ or *met16*Δ mutants (thus explaining their susceptibility phenotype). In mammalian cells a similar hypothesis has been raised based on the idea that synthesis of cysteine and cystine (the oxidized form of cysteine) can serve as a scavenging mechanism for excess sulfite [41]. Further studies are required to test this hypothesis and mechanistically understand what is the precise role of the sulfate activating pathway in SO_2_ detoxification by yeast cells, although it seems clearer that it goes beyond the conversion of exceeding sulfite into sulfide.

The involvement of Com2 in response and tolerance of *S. cerevisiae* cells to stress induced by SO_2_ at low pH is the first biological function attributed to this regulator. Transcriptomic analysis revealed that Com2 regulates, directly or indirectly, approximately 80% of the SO_2_-activated genes. According with the information available in the YEASTRACT database [42], this percentage of targets is much higher than the one predicted for other stress-responsive transcription factors (e.g. Msn2/Msn4 had only 47% of documented targets among the SO_2_-activated genes; Figure S6 and data not shown). These results clearly highlight Com2 as the main player in the reprogramming of yeast genomic expression under SO_2_ stress. In total, 47 genes activated by Com2 in response to SO_2_ were found to contribute for maximal tolerance to this chemical including not only the genes of the sulfate activating pathway but also genes required for lysine and arginine biosynthesis, among others that have more general functions in response to environmental stress. Consistently, wine fermentations undertaken in the presence of SO_2_ rapidly exhaust the arginine available in the must (comparing with the consumption observed in the absence of SO_2_) [43, 44], this also being observed for lysine although at a much less significant extent [43].

Most of the SO_2_-activated genes that are documented targets of Msn2 were also found to be regulated by Com2 suggesting some overlap between these two regulatory systems (Figure S6). The DNA sequence recognized by Com2 is not yet known, however, the C-terminal region of this regulator is highly similar to the DNA binding domains of ScMsn2 and CaMnl1 (Figure S7) which prompted us to search the promoter region of Com2-targets for STRE-like motifs (5'-CCCCT-3'=), known to serve as binding sites for ScMsn2 and CaMnl1 [26]. A high percentage of the SO_2_-induced genes identified as targets of Com2 and Msn2 harbor in their promoter region STRE-like sequences (Figure S6), however, these were not abundant on the promoters of the genes specifically under the regulation of Com2 (Figure S6). This leads us to conclude that either Com2 recognizes a STRE-like sequence and the effect observed on the regulation of genes only regulated by this transcription factor is indirect and mediated via other regulators; or Com2 recognize a sequence less specific than STRE.

In the absence of SO_2_ Com2 had no significant effect on the yeast transcriptome suggesting that it becomes active only when cells are exposed to this chemical. In line with this, up to now only *IME1* had been identified as a Com2 target [45] regardless the extensive number of studies describing transcriptional reprogramming of yeast cells under various stress conditions. Recently, several yeast stress-responsive transcription factors have been found to become activated upon binding of xenobiotics this being reported, for example, for the drug-induced regulator Pdr1 [46] as well as for Haa1 that was found to directly bind acetate [47]. Further studies are required to demonstrate if Com2 becomes activated in response to SO_2_ as the result of binding sulfite or metabisulfite. A shift in cytoplasmic to nuclear localization may also be another hypothesis as a regulatory mechanism for Com2 activity, considering that this was described to regulate ScMsn2 activity [48–51], however, the localization signals for nuclear import (NLS) and export (NES) mapped in Msn2 (between 575-642 residues) [52] are not conserved in Com2 (Fig. S7) and no other NLS or NES can be bioinformatically predicted for this regulator (Figure S7). Although Com2 had been identified as the orphan-homologue of *C. albicans* Mnl1 they do not appear to respond to the same stimuli since Com2 is dispensable for tolerance and response to acetic acid [27, 29], while Mnl1 is essential for this response [26]. Furthermore, no significant over-representation of genes of the Mnl1-regulon was observed in a recent survey of *C. albicans* response to sulfite stress [33].

A model of how *S. cerevisiae* cells respond at a genome-wide level to SO_2_-induced stress is proposed in **Fig. 6?** integrating the data that we have gathered in our work with previously established knowledge. The central role of Com2 in SO_2_ response and tolerance is highlighted in this scheme **(Fig. 6?)**, showing its involvement in the up-regulation of newly uncovered determinants of resistance like the genes of the sulfate activating pathway or the genes required for biosynthesis of lysine and arginine. It is possible that SO_2_-induced stress can lead to a depletion of intracellular lysine and arginine which can justify the protective effect exerted by enzymes contributing to the synthesis of these two amino acids as well, however, further studies are required to clarify this. Interestingly, recently it was shown that an increase in intracellular arginine improves yeast tolerance to inhibitory concentrations of ethanol by contributing to the integrity of the cell wall and the plasma membrane [53], which are also two well-described targets of SO_2_-induced toxicity according to the results of our chemogenomics screening. Notably, while supplementation of the MMB medium with lysine led to improved growth in the presence of SO_2_, no significant effect was obtained upon arginine supplementation which can result from a difficulty in increasing the intracellular concentration of this amino acid just by increasing its availability in the growth medium since the arginine uptake is complex and involves many players whose regulation of activity can be affected by the experimental conditions, including the exposure to SO_2_. Nonetheless, the demonstration that lysine supplementation can alleviate the toxic effects of SO_2_ in *S. cerevisiae* represents an important finding of our work since it can be easily explored by winemakers to improve performance of yeast strains along vinification, especially those that are more susceptible to the preservative action of SO_2_.

It is expected that the knowledge gathered in this work can be used for the development of more efficient wine preservation strategies based on SO_2_, either helping to understand what can be the molecular mechanisms behind the highly tolerant spoilage yeasts *B. bruxellensis* or S*. ludwigii* or by guiding the identification of what are the adaptive responses necessary to increase robustness of *S. cerevisiae* strains to be used in vinification. The herein identified set of genes involved in response and resistance to SO_2_ may also pave the way for a better understanding of what can be the toxicity mechanisms exerted by this preservative for humans in a more toxicological perspective.

## MATERIALS AND METHODS

### Strains and growth media

The parental strain *Saccharomyces cerevisiae* BY4741 (MATa, his3Δ1, leu2Δ0, met15Δ0, ura3Δ0) and the 19 individual single deletion mutant strains (*yer130cΔ, msn2Δ, msn4Δ, haa1Δ, met3Δ, met14Δ, met16Δ, met5Δ, met10Δ, arg2Δ, arg3Δ, arg4Δ, arg5,6Δ, arg7Δ, arg8Δ, lys4Δ, lys21Δ, lys14Δ* and *lys20Δ*) used in this work were all acquired from Euroscarf. The prototrophic strain *S. cerevisiae* yfg*Δ*0 (MATα::KanMX can1*Δ*::STE2prΔSpHIS5 *his3Δ1 lyp1Δ0*) as well as the derived mutants *met14Δ, met16Δ* and *met5Δ* were kindly provided by Dr. Amy Caudy [54]. The different yeast strains were cultivated in YPD medium (containing, per liter, 20g glucose (Merck), 10 g bactopeptone (HiMedia) and 5 g yeast extract (HiMedia)) or in minimal? medium MMB (containing, per liter, 1.7 g YNB without amino acids or ammonium sulfate (Difco Laboratories, Detroit, Michigan), 20 g glucose (Merck) and 2.65 g (NH_4_)_2_SO_4_ (Merck)). To surpass the auxotrophies of the BY4741 background, the MMB medium was further supplemented with 20 mg/L methionine, 60 mg/L leucine, 20 mg/L histidine, 20 mg/L uracil, all acquired from Sigma (Spain). Whenever required the pH of the MMB medium was adjusted to 3.5 using HCl as the acidulant. Preparation of the corresponding solid YPD or MMB media was achieved upon supplementation with 2% agar (Merck).

### Sulfur dioxide susceptibility assays

Susceptibility to SO_2_ of the parental strain BY4741 or of the selected deletion mutants was compared using spot assays or through the comparison of growth curves in liquid medium. For the spot assays, mid-exponential phase cells (OD_600nm_ ~ 0.6) cultivated in liquid MMB medium (at pH 3.5) at 30°C with orbital agitation (250 rpm), were used to prepare suspensions (in distilled water) having an OD_600nm_ of 0.1. These suspensions and two subsequent dilutions (1:5 and 1:10) were applied as spots (4 μl) onto the surface of solid MMB (pH 3.5) supplemented with SO_2_ concentrations ranging from 0 to 1.5 mM. SO_2_ was included in media by incorporating equal amounts of freshly prepared stock solutions of potassium metabisulfite (Merck) in water, with pH adjusted to 3.5, to give the desired final concentrations. Plates were incubated at 30°C for 2 to 3 days, depending on the severity of growth inhibition. For the comparison of the growth curves in liquid medium, mid-exponential phase cells (OD_600nm_ ~ 0.6) cultivated in MMB medium (at pH 3.5) were used to inoculate fresh medium at an initial OD_600nm_ of 0.2. Growth was followed by accompanying the increase in OD_600nm_ of the culture as well as the concentration of viable cells (assessed as the number of colony forming units per ml of cell culture (CFU ml^-1^) onto YPD solid medium, after 2 days of incubation at 30°C. Alternatively, growth of the wild-type and of the selected mutants in the presence or absence of SO_2_ was accompanied in 96-multiwell plates for 24h. The experimental setting used in this case was identical to the one described above with the exception that cultures were incubated in a plate reader (Multiskan Ascent spectrophotometer (Thermo Fisher Scientific Inc., Waltham, MA, USA)) and OD_600nm_ readings were taken every 30 min. All experiments were carried out in, at least, triplicates. To test the effect of lysine and arginine supplementation (1 g/L) cells were cultured in 96-multiwell plates as described above.

### Transcriptomic analysis of *S. cerevisiae* BY4741 and BY4741_*com2*Δ in response to SO_2_ stress

*S. cerevisiae* BY4741 and the mutant devoid of *COM2* were cultivated in MMB growth medium (at pH 3.5) until mid-exponential phase (standard OD_600nm_ 0.6) and then re-inoculated in fresh medium, either or not supplemented with 0.5 mM of SO_2_ (at pH 3.5). After 60 min of incubation in the absence or presence of SO_2_, cells were harvested, immediately frozen in liquid nitrogen, and kept at – 80°C until total RNA extraction. Total RNA extraction was performed according to the hot phenol method. Concentration and purity were determined by spectrophotometry and integrity was confirmed using an Agilent 2100 Bioanalyzer with a RNA 6000 Nano Assay (Agilent Technologies, Palo Alto, CA, USA). RNA was processed for use on Affymetrix (Santa Clara, CA, USA) GeneChip Yeast Genome 2.0 Arrays, according to the manufacturer's One-Cycle Target Labeling Assay. Briefly, 5 mg of total RNA containing spiked in Poly-A RNA controls (GeneChip Expression GeneChip Eukaryotic Poly-A RNA Control Kit; Affymetrix) was used in a reverse transcription reaction (One-Cycle DNA synthesis kit; Affymetrix) to generate first-strand cDNA. After second strand synthesis, double-stranded cDNA was used in an in vitro transcription (IVT) reaction to generate biotinylated cRNA (GeneChip Expression 30-Amplification Reagents for IVT-Labeling; Affymetrix). Size distribution of the cRNA and fragmented cRNA, respectively, was assessed using an Agilent 2100 Bioanalyzer with a RNA 6000 Nano Assay. A total of 5 μg of fragmented cRNA was used in a 100-μL hybridization cocktail containing added hybridization controls. 80 μl of mixture was hybridized on arrays for 16 h at 45 °C. Standard post hybridization wash and double-stain protocols (FS450_0003; GeneChip HWS kit) were used on an Affymetrix GeneChip Fluidics Station 450. Arrays were scanned on an Affymetrix Gene-Chip scanner 3000 7G.

The scanned arrays were analyzed first with Affymetrix MAS 5.0 software to obtain Absent/Present calls and subsequently with DNA-Chip Analyzer (dChip) 2010 (http://www.dchip.org, Wong Lab, Harvard). The arrays were normalized to a baseline array with median CEL intensity by applying an Invariant Set Normalization Method [40]. Normalized CEL intensities of the eight arrays were used to obtain model-based gene expression indices based on a PM (Perfect Match)-only model [40]. Microarray data are available from Gene Expression Omnibus (GEO) with accession number GSE 117883. Genes were considered to be differentially expressed if they were called Present in at least two replicates of each sample. A lower-confidence bound fold change cut-off between experiments and baseline was above 1.2, maintain a false discovery rate (FDR) below 10%. For downstream analysis of SO_2_-responsive genes only the genes whose transcriptional change was above or below 2-fold (p-value below 0.05) the values registered in control cells were considered to be altered. Clustering of the up- or down-regulated genes, based on biological function, was performed using the MIPS Functional Catalogue and the description of gene function is based on the information available in *Saccharomyces* Genome Database. To confirm some of the results obtained in the microarray analysis carried out, the transcript levels of selected SO_2_-responsive genes were compared by real-time RT-PCR in BY4741 and *com2*Δ cells. The same experimental setting used for the microarray analysis was used. Synthesis of cDNA from total RNA samples was performed using the MultiscribeTM reverse transcriptase kit and the subsequent real time RT-PCR step was carried out using SYBR® Green reagents 7500 RT-PCR in a Thermal Cycler Block (Applied Biosystems, Bedford, MA, USA). Specific primers for the amplification of the selected genes and of the *ACT1* gene, used as an internal control, were designed using Primer Express Software (Applied Biosystems) (available upon request).

### Phenotypic screening of the yeast deletion mutant collection to SO_2_-induced stress

To screen the Euroscarf haploid yeast mutant collection the strains were grown for 16h in 96-wells plate in MMB medium (at pH 3.5) at 30°C with orbital agitation (250 rpm). Five replicates of the wild type strain BY4741 were included in each plate to minimize inter and intra experimental condition variation. Then, 3 μL of the cellular suspensions were spotted (using a 96-pin replica platter) onto the surface of MMB solid medium (at pH 3.5) supplemented with 0, 1 or 1.5 mM SO_2_. Yeast cells growth was visually inspected after incubation at 30°C during 3 days. Strains showing a growth defect in control plates, without SO_2_, were discarded. Strains showing reduced growth comparing to the one obtained for the wild-type strain in the presence of 1 mM SO_2_ were considered highly susceptible, while those showing a growth defect only in the presence of 1.5 were considered susceptible.

### Assessment of intracellular reactive oxygen species in SO_2_-challenged cells

Cells of the parental strain BY4741 were cultivated, at 30°C with orbital agitation (250 rpm), in MMB (pH 3.5) until exponential-phase and then re-inoculated into this same fresh medium supplemented with 0 (control) or 0.5 mM of SO_2_ (at pH 3.5). After 200 minutes of incubation at 30 °C (maintaining an orbital agitation of 250 rpm) cells were stained with dihydroethidium (DHE, Molecular Probes, Eugene, USA) or with dihydrorhodamine 123 (DHR123, Molecular Probes, Eugene, USA) to determine intracellular reactive oxygen species (ROS), as described in (Mesquita A et al., 2010). DHE (10 μg ml^-1^) was added to the cell suspensions and these were further incubated for 10 min in dark. For the DHR123 staining, 15 μg ml^-1^ of DHR123 were added and cells incubated for 90 minutes at 30°C in the dark. Fluorescence emitted by DHE or DHR123 was analyzed by LSRII flow cytometer (BD-Biosciences) with a 488 nm excitation laser. The DHE and DHR123 signals were collected through a 488-nm blocking filter, with a 685-nm long-pass or a 505-nm long-pass, respectively. 30,000 cells/sample were captured at a low flow rate of 1,000 cells/s. Data collected with the LSRII flow cytometer were processed with Flowjo software (Tree Star).

## SUPPLEMENTAL MATERIAL

Click here for supplemental data file.

All supplemental data for this article are available online at http://www.microbialcell.com/researcharticles/2019a-lage-microbial-cell/.

## Abbreviations:

ROS: – reactive oxygen species.
