# Supplementary material for: Transcriptomic and chemogenomic analyses unveil the essential role of Com2-regulon in response and tolerance of Saccharomyces cerevisiae to stress induced by sulfur dioxide
Source: Microb Cell. 2019 Sep 30;6(11):509–23. doi: 10.15698/mic2019.11.697 (PMC6859422; doi:10.15698/mic2019.11.697)
Supplement: Supplementary file 1 [file mic-06-509-s01.pdf]

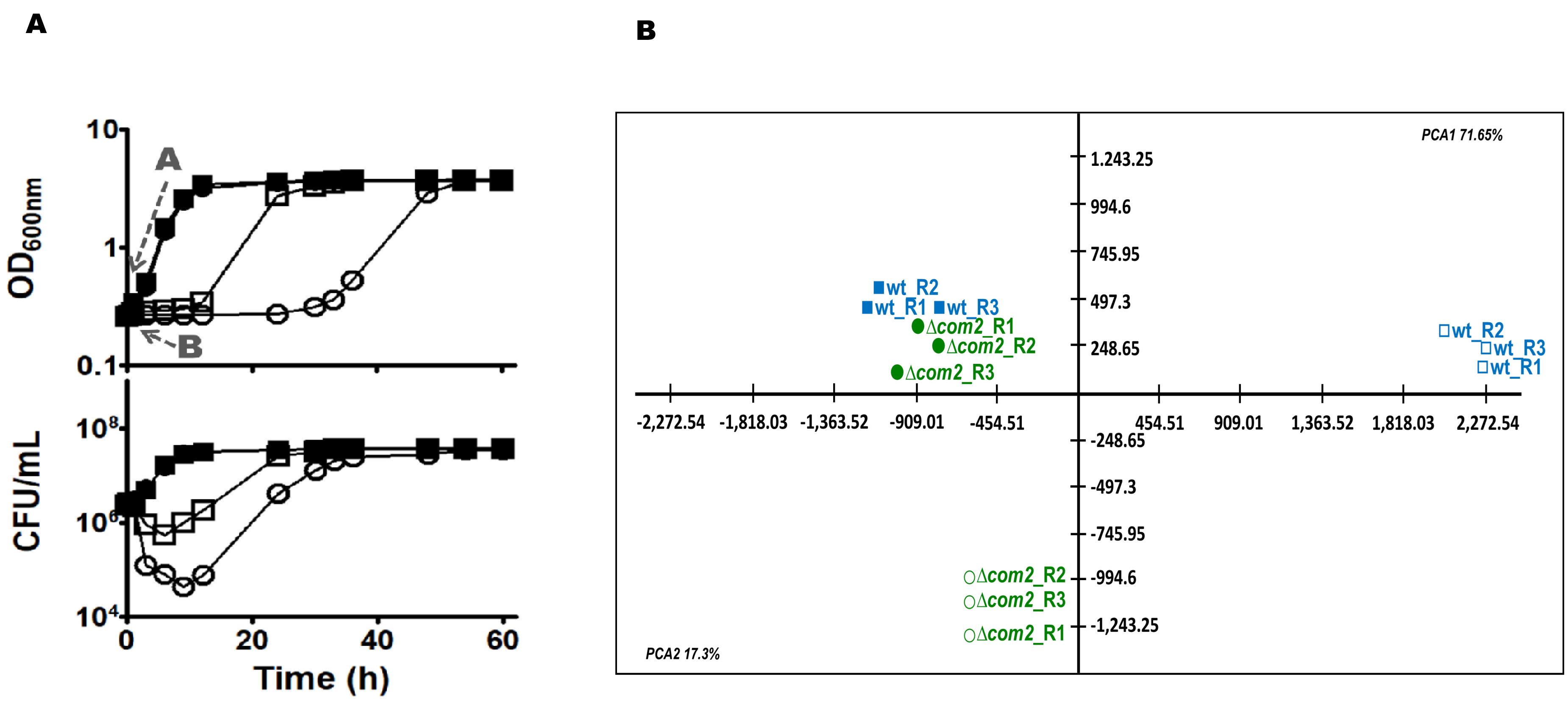

Supplementary Fig. S1 - (**A**) Growth curves of *S. cerevisiae* BY4741 (■, □) and of the deletion mutant *Δcom2* (●, ○) in MMB medium (pH 3.5) (■, ●) or in the same basal medium supplemented with 0.5mM SO_2 (pH 3.5) (□, ○). The arrows indicate the time of cultivation at which cell sample were harvested to compare the transcriptomes. Growth was followed by measuring culture $OD6_{00nm}$ and the concentration of viable cells was assessed as the number of colony-forming units/mL of cell culture (CFU mL^{-1}). The growth curves shown are means of, at least, three independent growth curves that gave rise to the same growth pattern. (**B**) PCA analysis of global transcriptional response, showing variation in expression levels between the parental strain *S. cerevisiae* BY4741 (blue) and in the *Δcom2* mutant (green), in cells untreated (filled marks) or treated with 0.5 mM SO_2 (open marks).

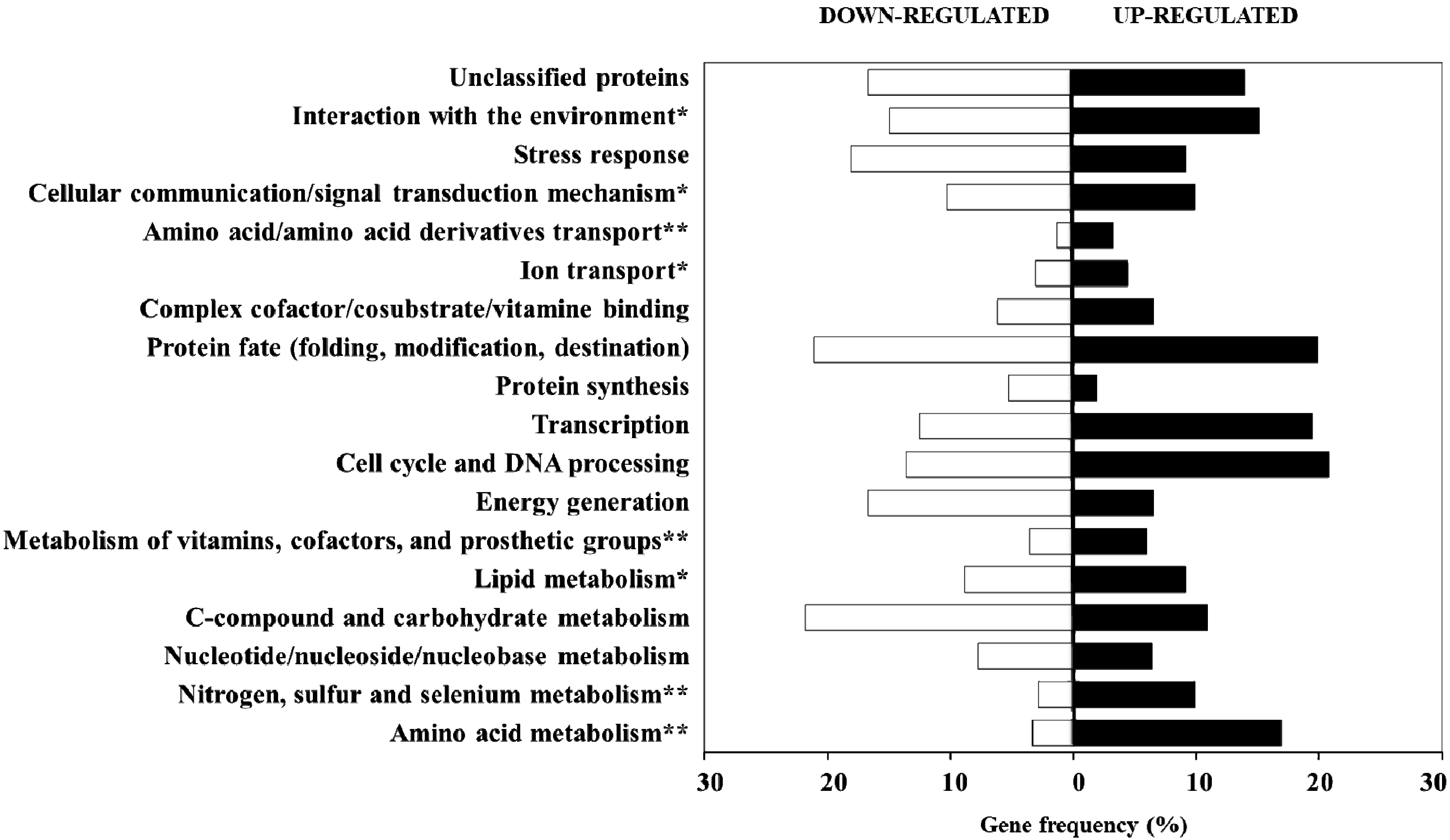

Supplementary Fig. S2 – Functional clustering, of genes up- (black bars) or downregulated (white bars) in S. cerevisiae BY4741 cells cultivated for 60 min in the presence of 0.5 mM sulphur dioxide (at pH 3.5). The classification into the different classes was performed using MIPS functional catalogue and the enriched functional classes (those having an associated p-value below 0.01) are marked with *.

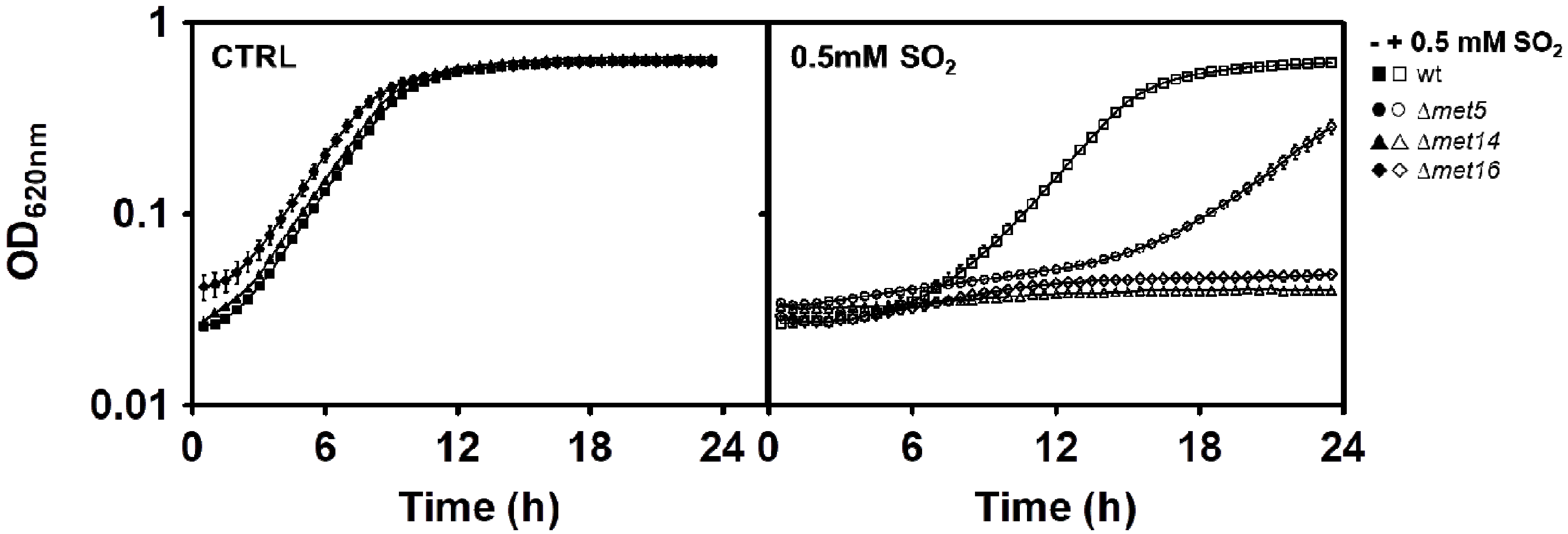

Supplementary Fig. S3 - Comparison of the susceptibility towards SO_2 of the prototrophic *Saccharomyces cerevisiae* yfgΔ0 strain and of the derived mutants *Δmet14*, *Δmet16* and *Δmet5*. Cells of the parental strain (wt) and of the indicated deletion mutants were grown until mid-exponential phase in liquid MMB medium (at pH 3.5) and then used to inoculate the same basal medium either supplemented (open symbols) or not supplemented with 0.5 mM SO_2 (filled symbols) (at pH 3.5). Cells were batch cultured at 30ºC and growth was monitored based on OD_{620nm}. The growth curves presented are representative of at least three independent growth experiments.

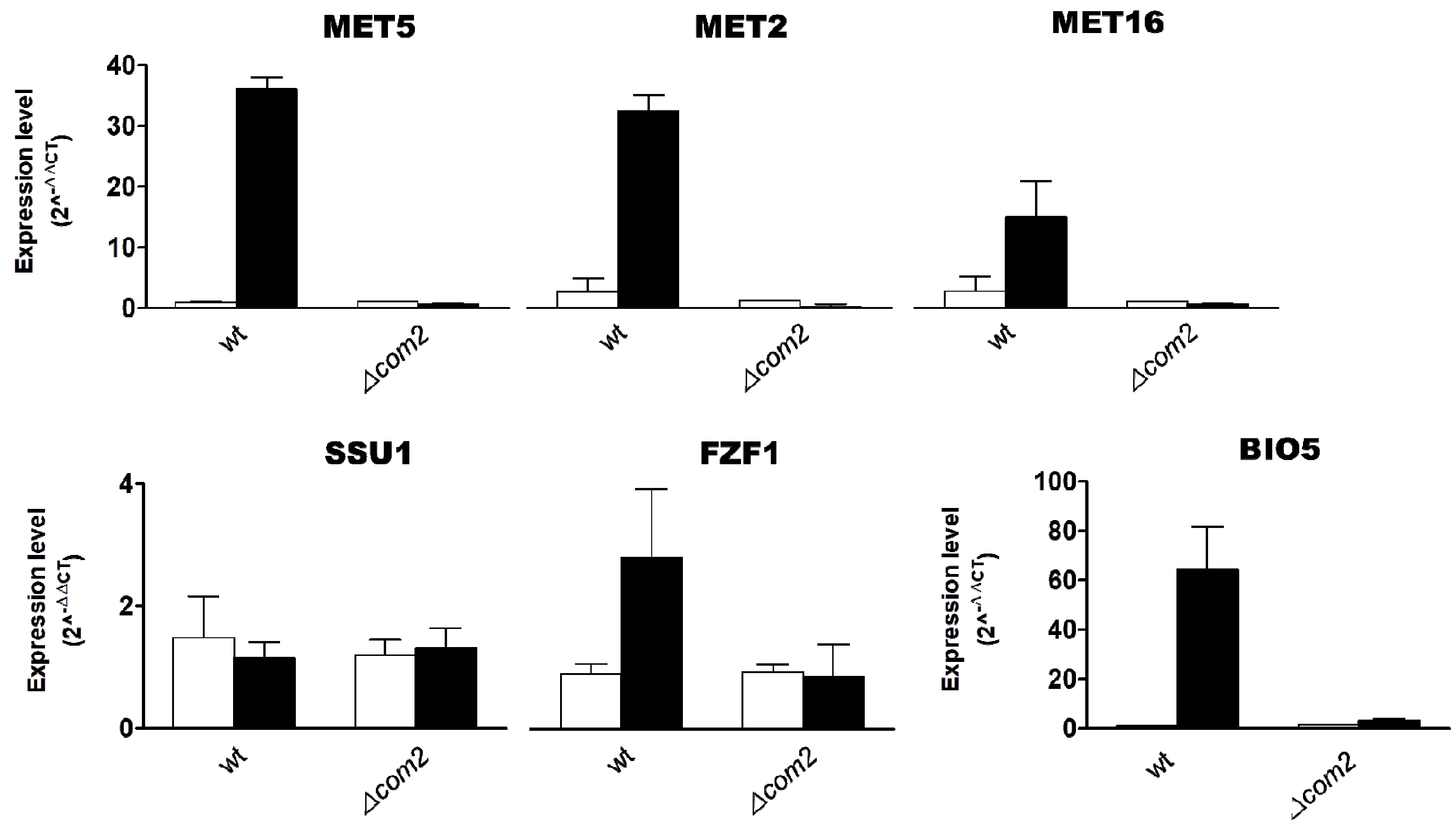

Supplementary Fig. S4 - Comparison of the transcript levels, estimated by real-time RT-PCR, of MET5, MET2, MET16, BIO5, SSU1 and FZF1 genes produced in cells of the parental strain BY4741 or the *Δcom2* mutant during cultivation for 60 min in MMB growth medium (at pH 3.5) unsupplemented (white bars) or supplemented with 0.5mM SO_2 (black bars). The expression of each gene determined by real-time qRT-PCR was normalized to the expression of the house-keeping gene, *ACT1* (encoding the structural protein actin).

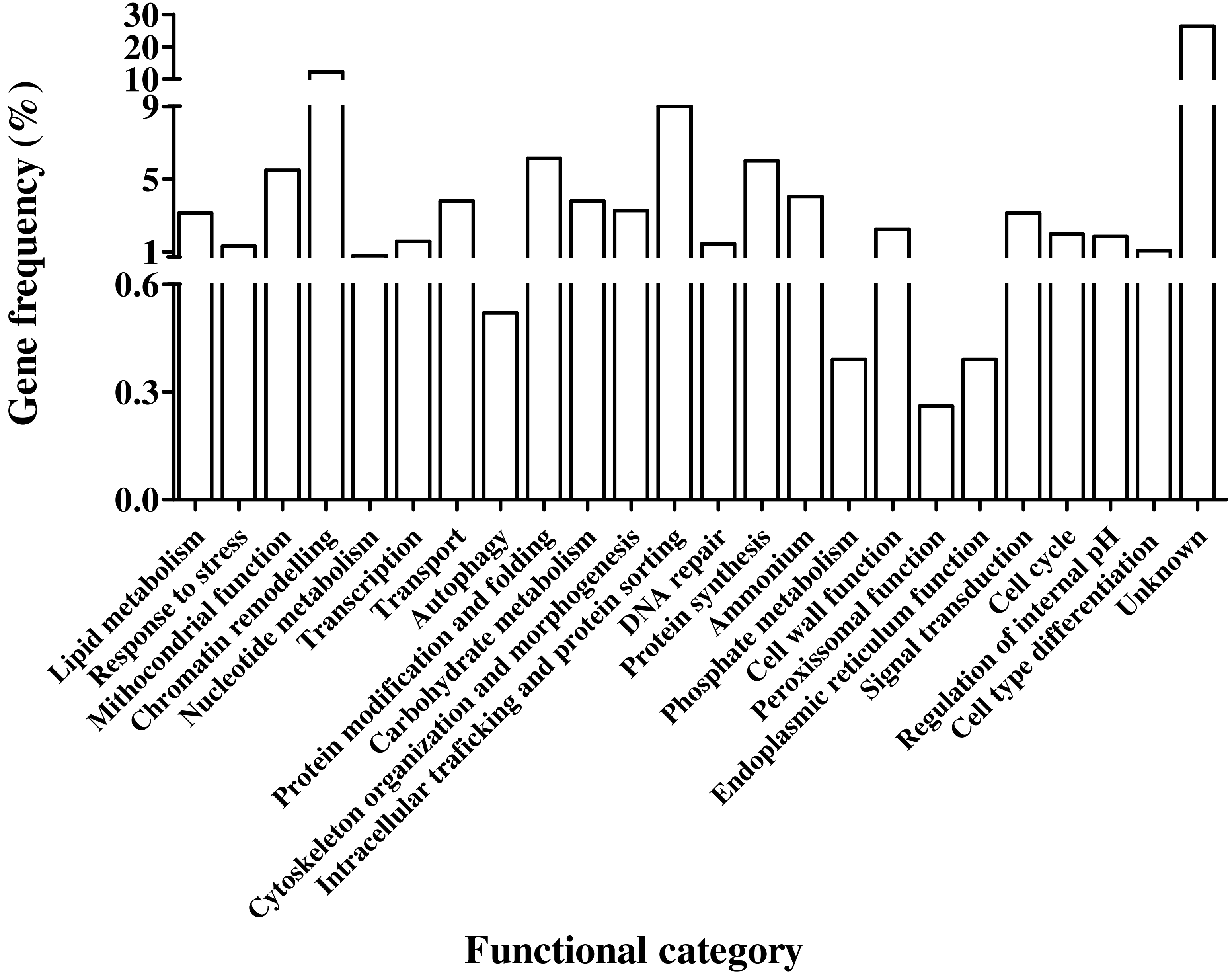

Supplementary Fig. S5 – Functional categorization of genes whose deletion lead to increased sensitivity to 0.5, 1 or 1.5 mM of SO_2 (at pH 3.5). For each functional class the frequency of genes present in our dataset of SO_2-resistance genes is shown.

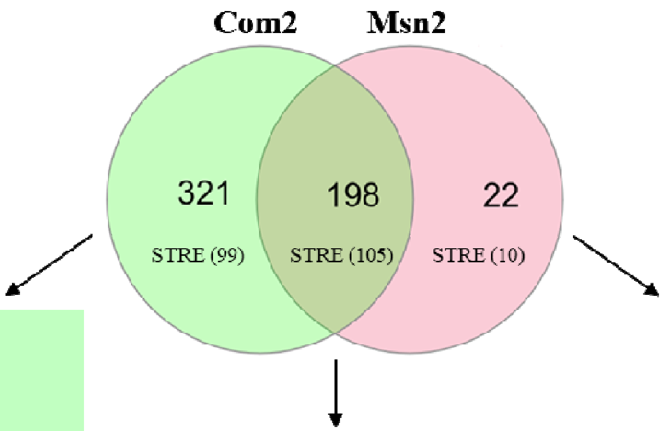

Sulphate assimilation pathway
MET8, MET10, MET3, MET5, MET1, MET22

Biosynthesis of vitamins, cofactors, and prosthetic groups
RIB7, RIB5, THI3, LYS5, BIO2, PAN5, BNA3, FOL1, BIO5, BIO3, DFR1, POS5, YAH1, THI21

Biosynthesis of lysine
LYS21, LYS14, LYS4, LYS5

Biosynthesis of arginine
ARG5,6, ARG2, ARG7, ARG82, ORT1

C-compound and carbohydrate
CTP1, MCH1, ITR1, YAT2, ESBP6, SAM3

Transcriptional regulation
ZAP1, BAS1, ARG81, URE2, SWI5, MET32, OPI1, STP2, GZF3, PUT3, LEU3

Cell cycle
BUD14, BUD8, BUD16, CDC20, DBF2, DBF20, DMA2, WHI2, KIP2

Others
COG1, HIS7, IBA57, MOG1, SWD3, BDS1, EEB1, GGC1, NRM1, PKP1, SHR5, SNA4, VAC17, VPS41, VPS9, YER039C-A, YMR001C-A

Biosynthesis of leucine
LEU1, LEU4, LEU9

Regulation of amino acid metabolism
ARO8, ARO9, MET30, MET28, ARG80, MET4, ARG3, CPA1, NPR3

Biosynthesis of valine
ILV3, ILV5, ILV2

Transport
SEO1, YMC2, MDM34, YGL114W, VHT1, VHR1, ISA1, MCH4, MCH5, STP22

Catabolism of nitrogenous compounds
DAL3, DAL82

Others
FKS3, ICP55, MNN4, YOR302W, EHT1, INO2, PCL7, PHO5, RTC2, SIM1

Transport
UGA4, DUR3, CRC1

Others
IRC7, ZRT1, ECM13, PCL2, YDL183C, AMD2, ICL1, CLB1, RTS3, SPO20, YJL213W, YJR154W, YKL068W-A, ICY1, SNO4, MUM3, SFG1, DAL80

Supplementary Fig. S6 – Clustering of SO_2-activated genes with the transcription factors Msn2 and Com2, based on the results obtained in this study and in previously documented regulatory associations described in YEASTRACT database. The number of genes whose promoters harbor a STRE-motif (5'-CCCCT-3') is also indicated. Underlined genes are those genes conferring tolerance to SO_2, according with the results of the disruptome screening.

Supplementary Fig. S7 **-** Amino acid sequence alignment of ScMsn2, ScMsn4, ScCom2 and *Candida albicans* Mnl1. The picture depicts the similarity registered at the level of the DNA-binding domain of these three regulators (highlighted as DBD in the end of the alignment) as well as the lack of similarity registered in domain HD1 comprising the nuclear export signal of Msn2. The two mapped regions are according with the motifs mapping described by Pfanzagl et al., 2018. For the alignment, the software “Multiple Sequence Alignment Clustal Omega” was applied. Refinements were conducted with the software “Jalview.” The blue color intensity correlates with the degree of conservation.

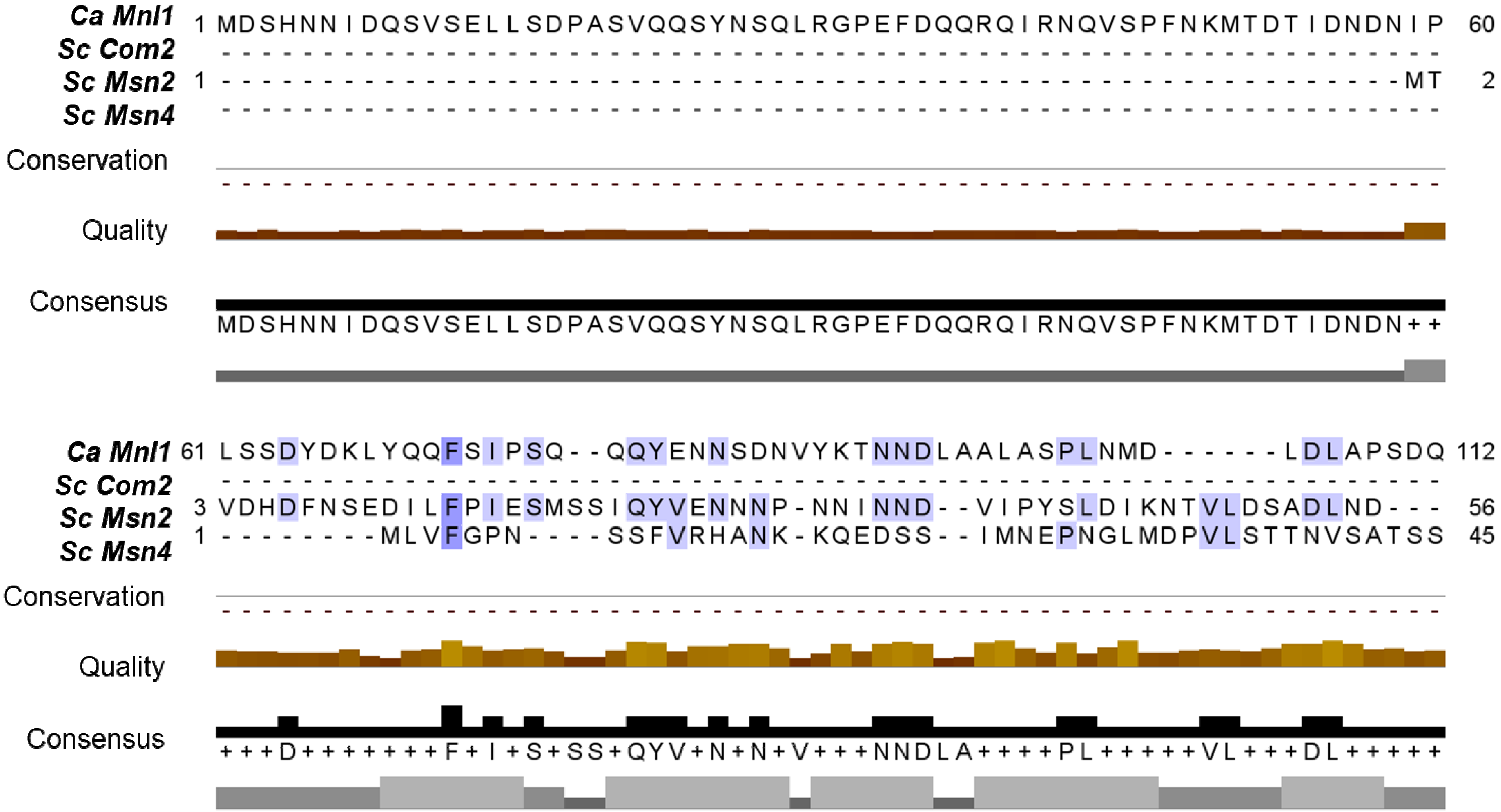

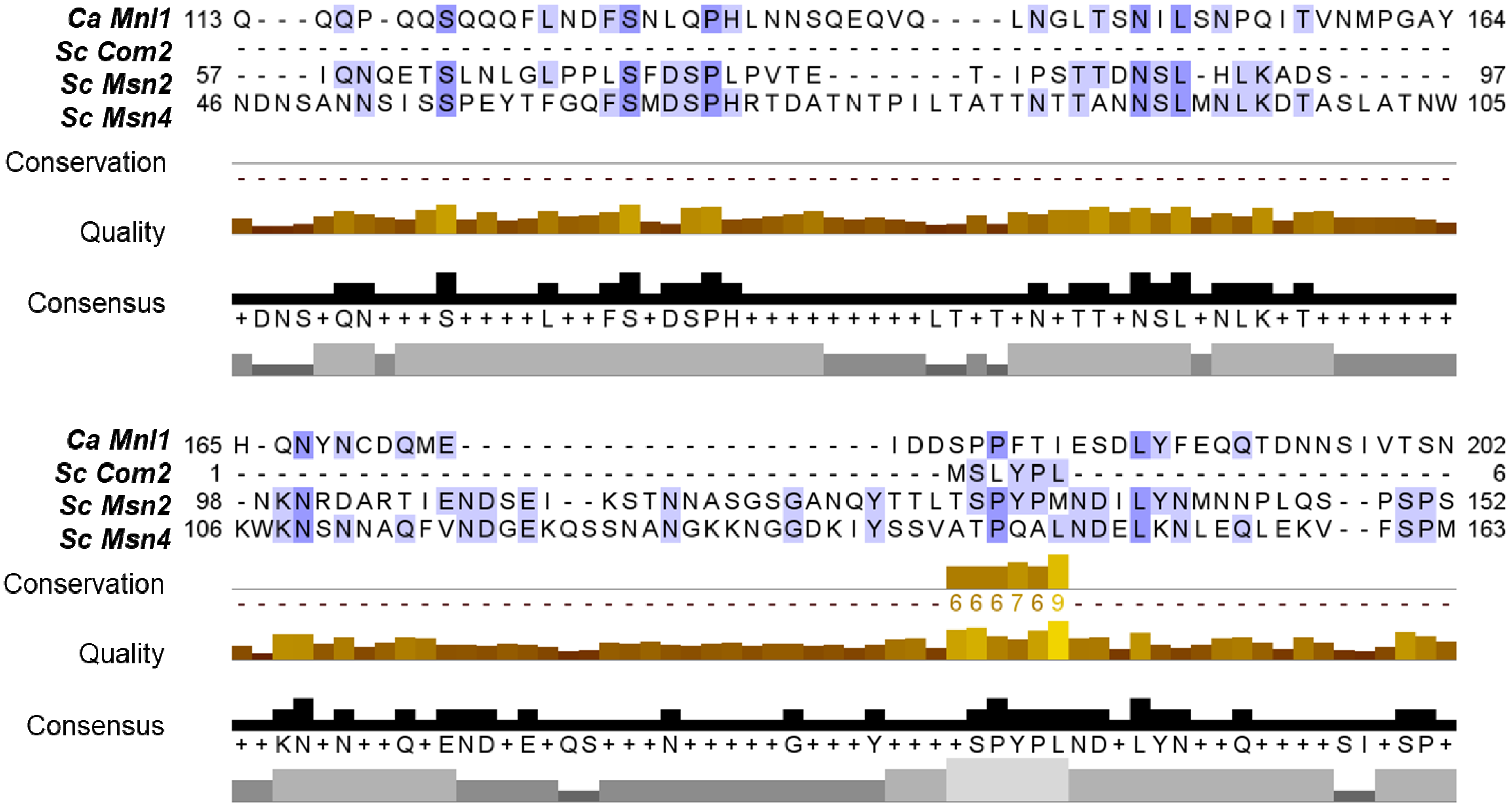

Ca Mnl1 113 Q---QQP-QQSQQQFLNDFSNLQPHLNNSQEQVQ----LNGLTSNILSNPQITVNMPGAY 164
Sc Com2 --
Sc Msn2 57 ----IQNQETSLNLGLPPLSFDSPLPVTE-------T-IPSTTDNSL-HLKADS------ 97
Sc Msn4 46 NDNSANNSISSPEYTFGQFSMDSPHRTDATNTPILTATTNTTANNSLMNLKDTASLATNW 105
Conservation
Quality
Consensus
+DNS+QN+++S++++L++FS+DSPH+++++++++LT+T+N+TT+NSL+NLK+T+++++++
Ca Mnl1 165 H-QNYNCDQME-------------------IDDSPPFTIESDLYFEQQTDNNSIVTSN 202
Sc Com2 1 ------------------------------MSLYPL---------------------- 6
Sc Msn2 98 -NKNRDARTIENDSEI--KSTNNASGSGANQYTTLTSPYPMNDILYNMNNPLQS--PSPS 152
Sc Msn4 106 KWKNSNNAQFVNDGEKQSSNANGKKNGGDKIYSSVATPQALNDELKNLEQLEKV--FSPM 163
Conservation
666769
Quality
Consensus
++KN+N++Q+END+E+QS+++N+++++G+++Y++++SPYPLND+LYN++Q++++SI+SP+

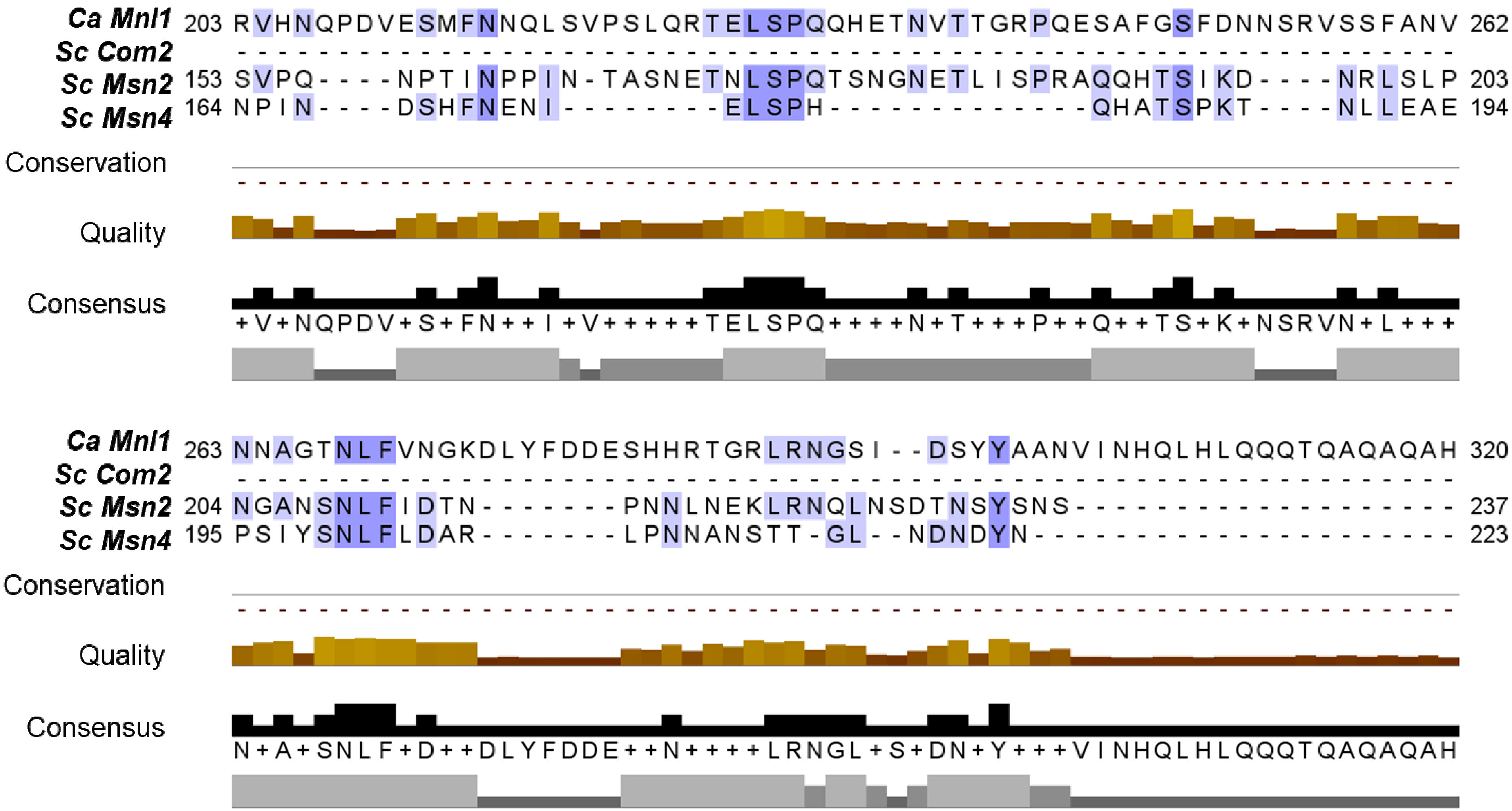
Ca Mnl1 203 RVHNQPDVESMFNNQLSVPSLQRTELSPQQHETNVTTGRPQESAFGSFDNNSRVSSFANV 262
Sc Com2 --
Sc Msn2 153 SVPQ----NPTINPPIN-TASNETNLSPQTSNGNETLISPRAQQHTSIKD----NRLSLP 203
Sc Msn4 164 NPIN----DSHFNENI--------ELSPH-------------QHATSPKT----NLLEAE 194
Conservation
Quality
Consensus
+V+NQPDV+S+FN++I+V+++++TELSPQ++++N+T+++P++Q++TS+K+NSRVN+L+++
Ca Mnl1 263 NNAGTNLFVNGKDLYFDDESHHRTGRLRNGSI--DSYYAANVINHQLHLQQQTQAQAQAH 320
Sc Com2 --
Sc Msn2 204 NGANSNLFIDTN-------PNNLNEKLRNQLNSDTNSYSNS------------------ 237
Sc Msn4 195 PSIYSNLFLDAR-------LPNNANSTT-GL--NDNDYN-------------------- 223
Conservation
Quality
Consensus
N+A+SNLF+D++DLYFDDE++N++++LRNGL+S+DN+Y+++VINHQLHLQQQTQAQAQAH

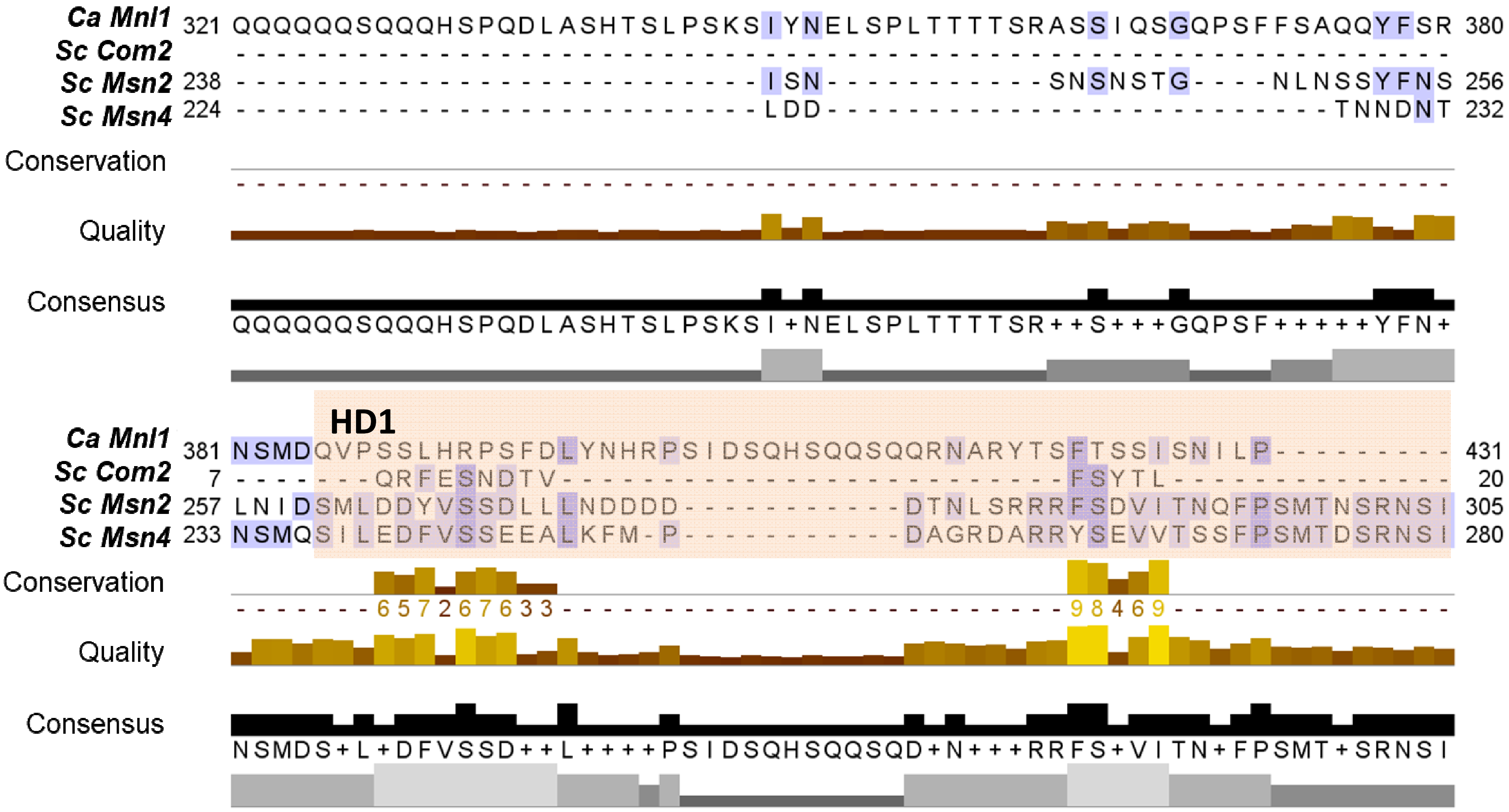

Ca Mnl1
Sc Com2
Sc Msn2
Sc Msn4
Conservation
Quality
Consensus
HD1

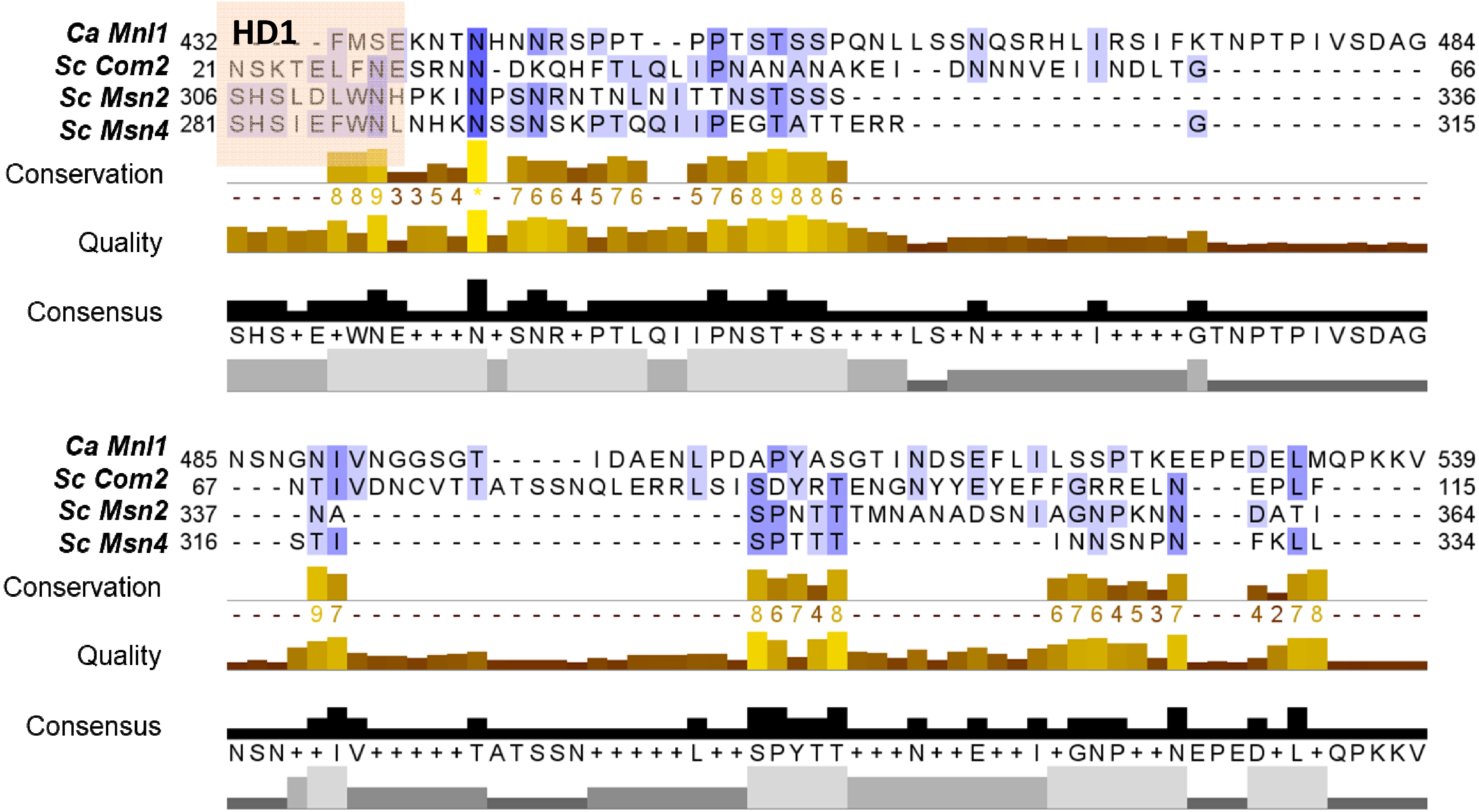
HD1
Ca Mnl1 432 FMSEKNTNHNNRSPPT--PPTSTSSPQNLLSSNQSRHLIRSIFKTNPTPIVSDAG 484
Sc Com2 21 NSKTELFNESRNN-DKQHFTLQLIPNANANAKEI--DNNNVEIINDLTG----------- 66
Sc Msn2 306 SHSLDLWNHPKINPSNRNTNLNITTNSTSSS 336
Sc Msn4 281 SHSIEFWNLNHKNSSNSKPTQQIIPEGTATTERR 315
Conservation
8893354*-7664576--5768986
Quality
Consensus
SHS+E+WNE+++N+SNR+PTLQIIPNST+S++++LS+N+++++I++++GTNPTPIVSDAG
Ca Mnl1 485 NSNGNIVNGGSGT-----IDAENLPDAPYASGTINDSEFLILSSPTKEEPEDELMQPKKV 539
Sc Com2 67 ---NTIVDNCVTTATSSNQLERRLSISDYRTENGNYYEYEFFGRRELN---EPLF------ 115
Sc Msn2 337 ----NA SPNTTTMNANADSNIAGNPKNN---DATI------ 364
Sc Msn4 316 ---STI SPTTT INNSNPN---FKLL------ 334
Conservation
97 86748 6764537 4278
Quality
Consensus
NSN++IV++++++TATSSN+++++L++SPYTT+++N++E++I+GNP++NEPED+L+QPKKV

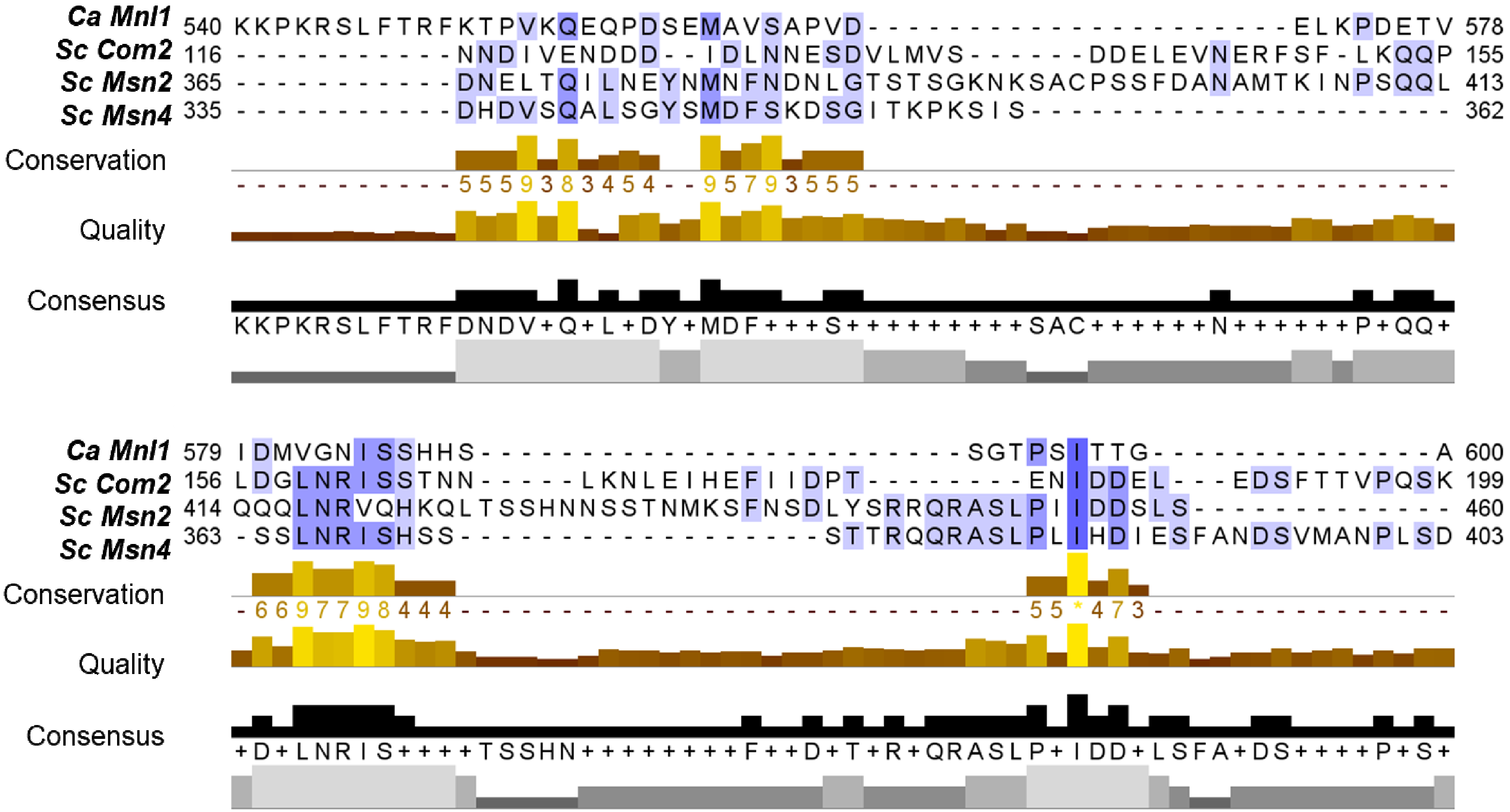

Ca Mnl1 540 KKPKRSLFTRFKTPVKQEQPDSEMAVSAPVD----------------------ELKPDETV 578
Sc Com2 116 -----------NNDIVENDDD--IDLNNESDVLMVS------DDELEVNERFSF-LKQQP 155
Sc Msn2 365 -----------DNELTQILNEYNMNFNDNLGTSTSGKNKSACPSSFDANAMTKINPSQQL 413
Sc Msn4 335 -----------DHDVSQALSGYSMDFSKDSGITKPKSIS---------------------- 362
Conservation
-----------5559383454--95793555----------------------
Quality
Consensus
KKPKRSLFTRFDNDV+Q+L+DY+MDF+++S+++++++++SAC++++++N++++++P+QQ+
Ca Mnl1 579 IDMVGNISSHHS------------------------SGTPSITTG--------------A 600
Sc Com2 156 LDGLNRISSTNN-----LKNLEIHEFIIDPT--------ENIDDEL---EDSFTTVPQSK 199
Sc Msn2 414 QQQLNRVQHKQLTSSHNNSSTNMKSFNSDLYSRRQRASLPIIDDSLS------------ 460
Sc Msn4 363 -SSLNRISHSS-------------------STTRQQRASLPLIHDIESFANDSVMANPLSD 403
Conservation
-6697798444------------------------55*473--------------
Quality
Consensus
+D+LNRIS++++TSSHN+++++++++F++D+T+R+QRASLP+IDD+LSFA+DS++++P+S+

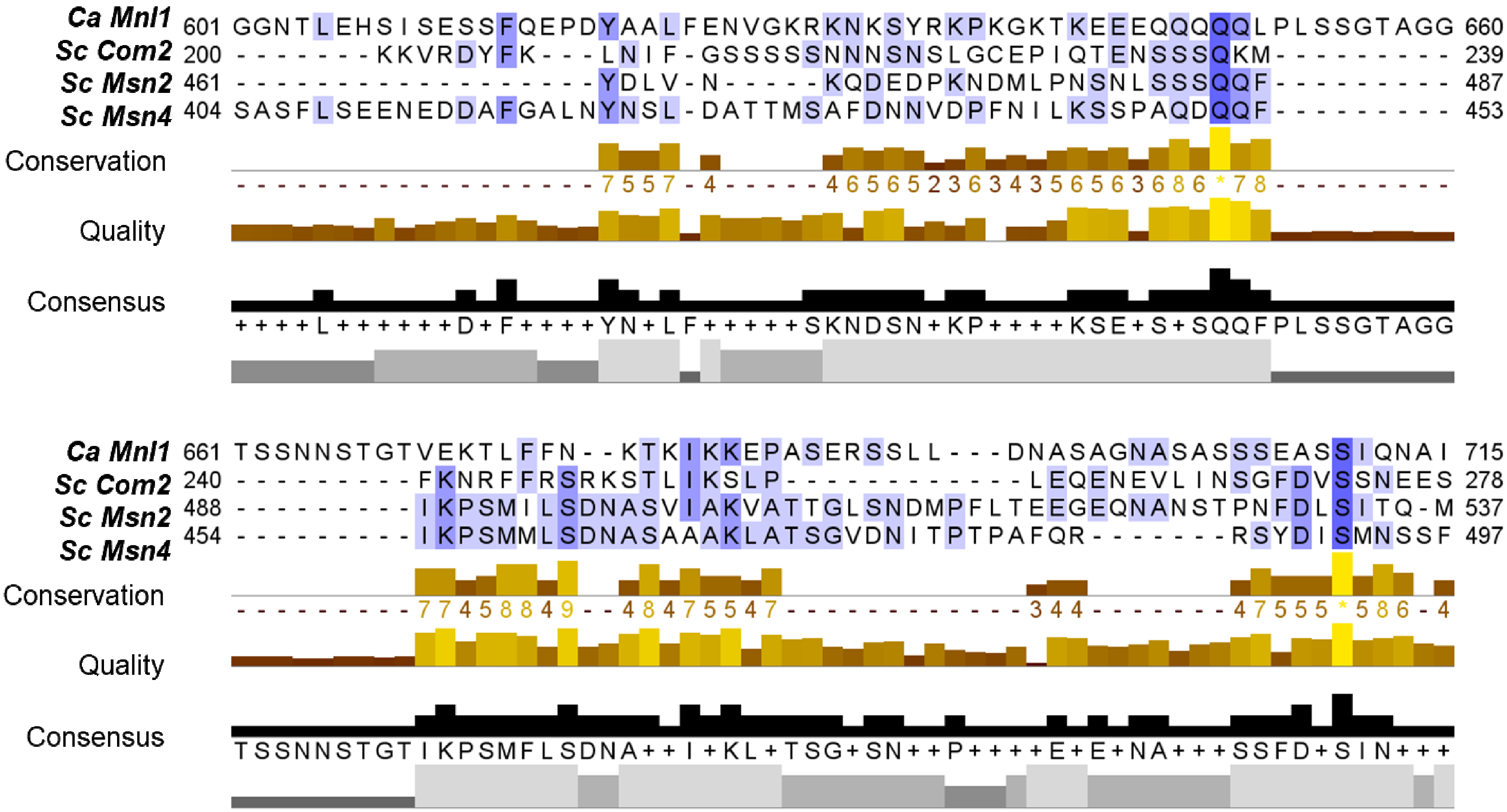
Ca Mnl1 601 GGNTLEHSISESSFQEPDYAALFENVGKRKNKSYRKPKGKTKEEEQQQQQLPLSSGTAGG 660
Sc Com2 200 --------KKVRDYFK---LNIF-GSSSSSNNNSNSLGCEPIQTENSSSQKM---------- 239
Sc Msn2 461 ------------------YDLV-N-----KQDEDPKNDMLPNSNLSSSQQF---------- 487
Sc Msn4 404 SASFLSEENEDDAFGALNYNSL-DATTMSAFDNNVDPFNILKSSPAQDQQF---------- 453
Conservation
------------------7557-4-----4656523634356563686*78---------
Quality
Consensus
++++L++++++D+F++++YN+LF+++++SKNDSN+KP++++KSE+S+SQQFPLSSGTAGG
Ca Mnl1 661 TSSNNSTGTVEKTLFFN--KTKIKKEPASERSSLL---DNASAGNASASSSEASSIQNAI 715
Sc Com2 240 ---------FKNRFFRSRKSTLIKSLP-----------LEQENEVLINSGFDVSSNEES 278
Sc Msn2 488 ---------IKPSMILSDNASVIAKVATTGLSNDMPFLTEEGEQNANSTPNFDLSITQ-M 537
Sc Msn4 454 ---------IKPSMMLSDNASAAAKLATSGVDNITPTPAFQR-------RSYDISMNSSF 497
Conservation
---------774588849--48475547-----------344--------47555*586-4
Quality
Consensus
TSSNNSTGTIKPSMFLSDNA++I+KL+TSG+SN++P++++E+E+NA+++SSFD+SIN+++

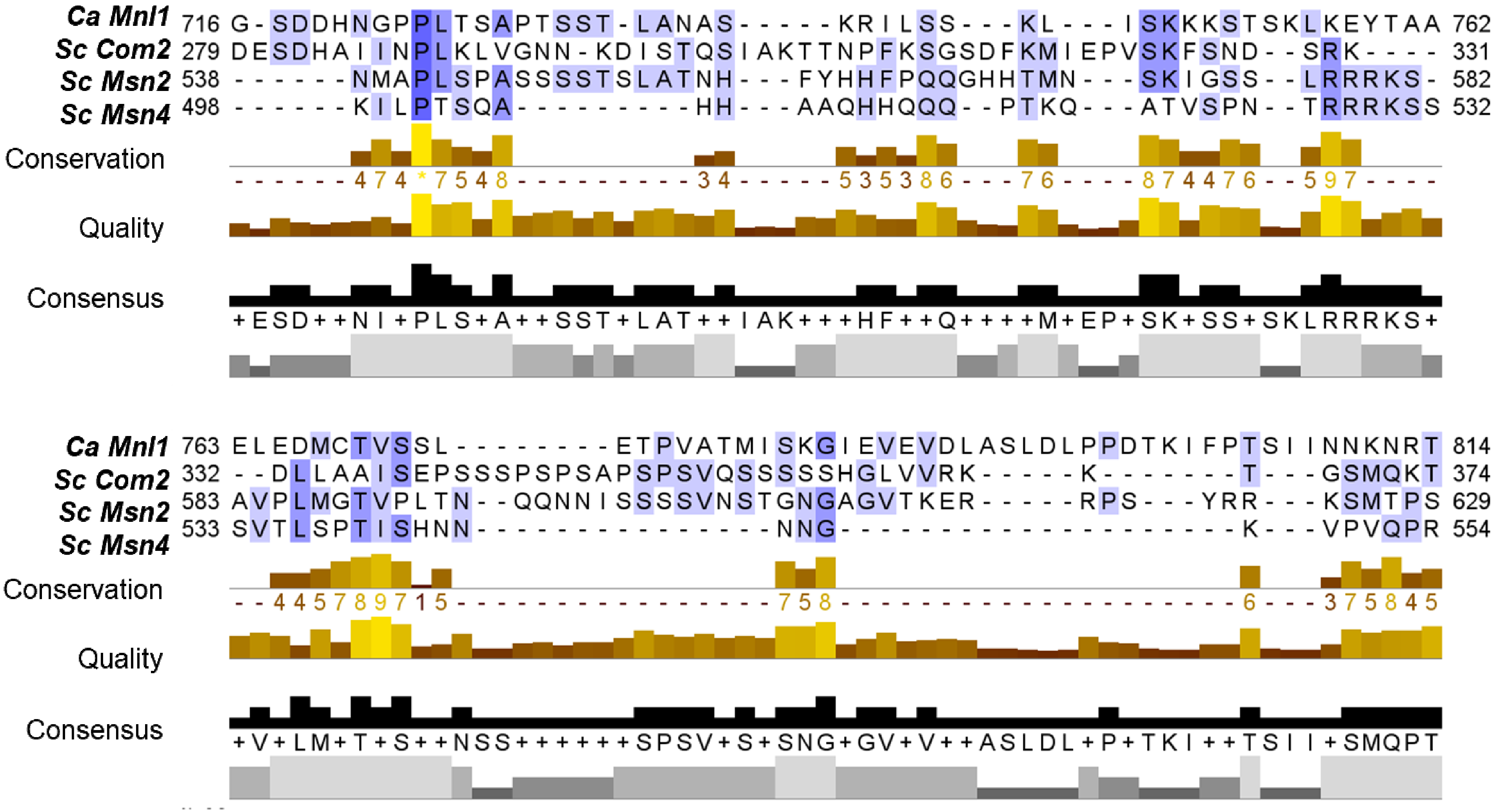

Ca Mnl1 716 G-SDDHNGPPLTSAPTSST-LANAS-----KRILSS---KL---ISKKKSTSKLKEYTAA 762
Sc Com2 279 DESDHAI INPLKLVGNN-KDISTQSIAKTTNPFKSGSDFKMIEPVSKFSND--SRK---- 331
Sc Msn2 538 ------NMAPLSPASSSSTSLATNH---FYHHFPQQGHHTMN---SKIGSS--LRRRKS- 582
Sc Msn4 498 ------KILPTSQA---------HH---AAQHHQQQ--PTKQ---ATVSPN--TRRRKSS 532
Conservation
------474*7548---------34-----535386---76----874476--597----
Quality
Consensus
+ESD++NI+PLS+A++SST+LAT++IAK+++HF++Q+++M+EP+SK+SS+SKLRRRKS+
Ca Mnl1 763 ELEDMCTVSSL--------ETPVATMISKGIEVEVDLASLDLPPDTKIFPTSIINNKNRT 814
Sc Com2 332 --DLLAAISEPSSSPSPSAPSPSVQSSSSSHGLVVRK-----K--------T---GSMQKT 374
Sc Msn2 583 AVPLMGTVPLTN--QQNNISSSSVNSTGNGAGVTKER-----RPS---YRR---KSMTPS 629
Sc Msn4 533 SVTLSPTISHNN----------------NNG---------------------K---VPVQPR 554
Conservation
--44578971 5----------------758------------------6---375845
Quality
Consensus
+V+LM+T+S++NSS++++++SPSV+S+SNG+GV+V++ASLDL+P+TKI++TSII+SMQPT

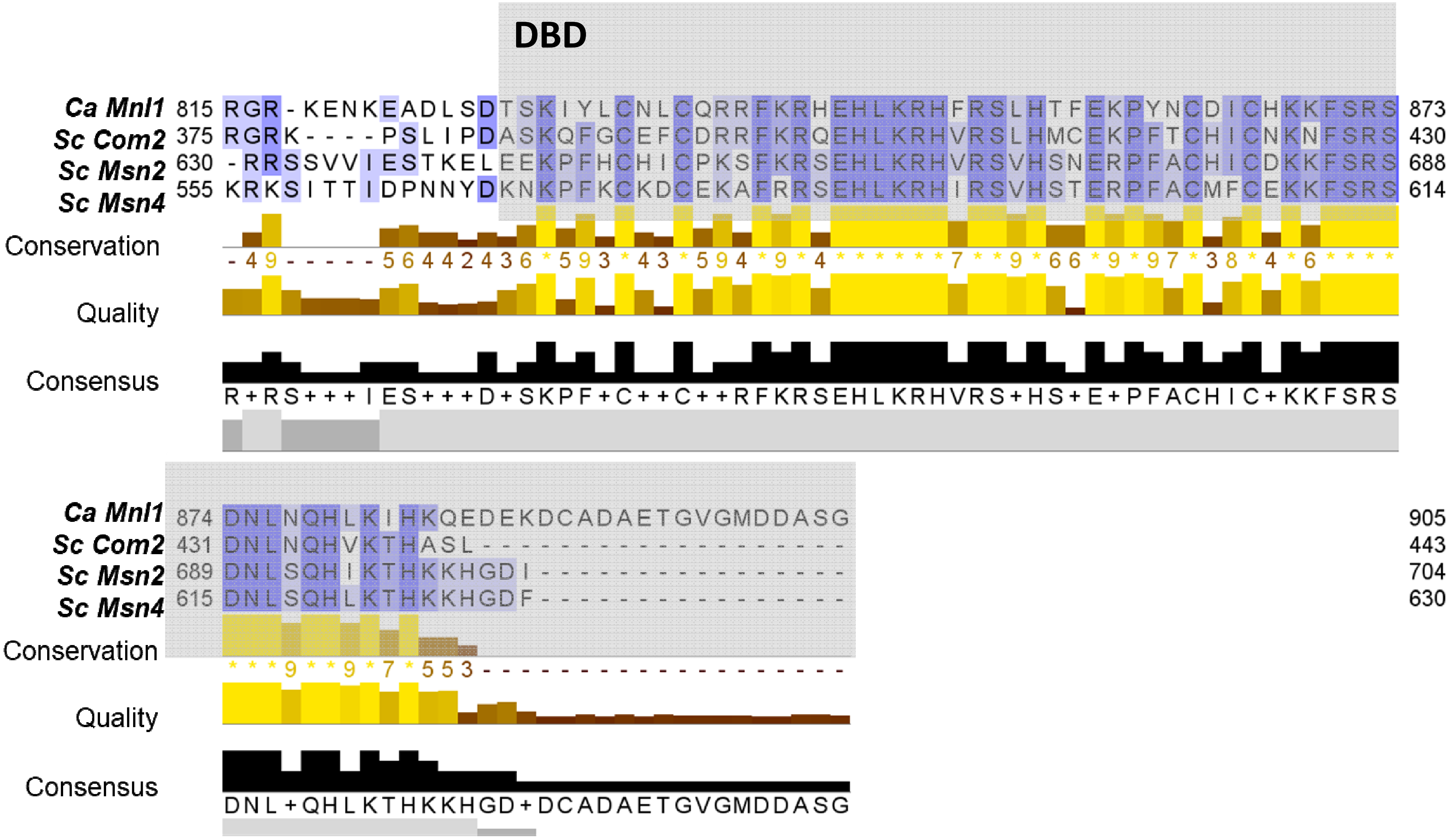
DBD
Ca Mnl1 815 RGR-KENKEADLSDTSKIYLCNLCQRRFKRHEHLKRHFRSLHTFEKPYNCDICHKKFSRS 873
Sc Com2 375 RGRK----PSLIPDASKQFGCEFCDRRFKRQEHLKRHVRSLHMCEKPFTCHICNKNFSRS 430
Sc Msn2 630 -RRSSVVIESTKELEEKPFHCHICPKSFKRSEHLKRHVRSVHSNERPFACHICDKKFSRS 688
Sc Msn4 555 KRKSITTIDPNNYDKNKPFKCKDCEKAFRRSEHLKRHIRSVHSTERPFACMFCEKKFSRS 614
Conservation
-49-----56442436*593*43*594*9*4******7**9*66*9*97*38*4*6****
Quality
Consensus
R+RS+++IES+++D+SKPF+C++C++RFKRSEHLKRHVRS+HS+E+PFACHIC+KKFSRS
Ca Mnl1 874 DNLNQHLKIHKQEDEKDCADAETGVGMDDASG 905
Sc Com2 431 DNLNQHVKTHASL------------------- 443
Sc Msn2 689 DNLSQHIKTHKKHGDI---------------- 704
Sc Msn4 615 DNLSQHLKTHKKHGDF---------------- 630
Conservation
***9**9*7*553-------------------
Quality
Consensus
DNL+QHLKTHKKHGD+DCADAETGVGMDDASG
